# Low eosinophil and low lymphocyte counts and the incidence of 12 cardiovascular diseases: a CALIBER cohort study

**DOI:** 10.1136/openhrt-2016-000477

**Published:** 2016-09-05

**Authors:** Anoop Dinesh Shah, Spiros Denaxas, Owen Nicholas, Aroon D Hingorani, Harry Hemingway

**Affiliations:** 1 Farr Institute of Health Informatics Research, UCL Institute of Health Informatics, University College London, London, UK; 2 University College London Hospitals NHS Foundation Trust, London, UK; 3 National Institute for Cardiovascular Outcomes Research, UCL Institute of Cardiovascular Science, University College London, London, UK

**Keywords:** HEART FAILURE

## Abstract

**Background:**

Eosinophil and lymphocyte counts are commonly performed in clinical practice. Previous studies provide conflicting evidence of association with cardiovascular diseases.

**Methods:**

We used linked primary care, hospitalisation, disease registry and mortality data in England (the CALIBER (CArdiovascular disease research using LInked Bespoke studies and Electronic health Records) programme). We included people aged 30 or older without cardiovascular disease at baseline, and used Cox models to estimate cause-specific HRs for the association of eosinophil or lymphocyte counts with the first occurrence of cardiovascular disease.

**Results:**

The cohort comprised 775 231 individuals, of whom 55 004 presented with cardiovascular disease over median follow-up 3.8 years. Over the first 6 months, there was a strong association of low eosinophil counts (<0.05 compared with 0.15–0.25×10^9^/L) with heart failure (adjusted HR 2.05; 95% CI 1.72 to 2.43), unheralded coronary death (HR 1.94, 95% CI 1.40 to 2.69), ventricular arrhythmia/sudden cardiac death and subarachnoid haemorrhage, but not angina, non-fatal myocardial infarction, transient ischaemic attack, ischaemic stroke, haemorrhagic stroke, subarachnoid haemorrhage or abdominal aortic aneurysm. Low eosinophil count was inversely associated with peripheral arterial disease (HR 0.63, 95% CI 0.44 to 0.89). There were similar associations with low lymphocyte counts (<1.45 vs 1.85–2.15×10^9^/L); adjusted HR over the first 6 months for heart failure was 2.25 (95% CI 1.90 to 2.67). Associations beyond the first 6 months were weaker.

**Conclusions:**

Low eosinophil counts and low lymphocyte counts in the general population are associated with increased short-term incidence of heart failure and coronary death.

**Trial registration number:**

NCT02014610; results.

**Key questions**

**What is already known about this subject?**

- Inflammation is a key pathological process underlying atherosclerosis and cardiovascular diseases, and different types of white blood cells (including eosinophils and lymphocytes) have specific roles in inflammation.
- It is not known how strongly eosinophil and lymphocyte counts in healthy populations are associated with the incidence of a wide range of pathologically diverse cardiovascular diseases. The few existing studies had a small number of participants or studied a limited range of cardiovascular diseases.

**What does this study add?**

- In a healthy population without pre-existing cardiovascular disease, low eosinophil and lymphocyte counts were associated with increased short-term incidence of heart failure, unheralded coronary death, ventricular arrhythmia/sudden cardiac death and non-cardiovascular death.
- These associations were specific; eosinophil and lymphocyte counts were not associated with incidence of angina, non-fatal myocardial infarction or stroke among people without pre-existing cardiovascular disease.

**How might this impact on clinical practice?**

- Clinicians should be aware that low eosinophil counts (in the range which is currently reported as ‘normal’) may be associated with increased short term risk of heart failure and death.
- Therapies which result in lower eosinophil counts, such as anti-interleukin-5 therapies for asthma, should be monitored for any increased risk of cardiovascular events in clinical trials and postmarketing surveillance.

## Introduction

Eosinophils and lymphocytes are two types of white blood cell (leucocyte). While other types of white blood cells such as neutrophils and monocytes are known to be involved in inflammatory processes that contribute to atherosclerosis,^1^ the role of eosinophils and lymphocytes in cardiovascular diseases is less well defined. Observational studies show worse prognosis among patients with heart failure with low eosinophil counts^2^ or low lymphocyte counts,^3–5^ prompting the question as to whether counts of these types of cell are also associated with onset of cardiovascular diseases in previously healthy people.

The main physiological role of eosinophils is thought to be in restoring tissue homoeostasis after inflammation.^6^ High eosinophil counts may be due to conditions such as asthma,^7^ but clinicians tend not to attribute particular significance to low or moderate eosinophil counts. Eosinophil counts increase acutely after myocardial infarction (MI)^8^ and large numbers are found in coronary thrombi.^9^ Two small cohort studies (Hiroshima and Nagasaki study^10^ and Caerphilly study^11^) found that higher eosinophil count was associated with greater incidence of coronary heart disease among healthy people (see online supplementary table S1). However, in patients undergoing percutaneous coronary intervention^12^ or those in critical care,^13^ lower eosinophil counts were associated with worse prognosis. The previous evidence relating eosinophil counts to cardiovascular diseases is thus scarce and contradictory.

10.1136/openhrt-2016-000477.supp1 Supplementary data

Lymphocytes comprise a range of different cell types with different roles in the immune system.^14^ Low total lymphocyte count is associated with malnutrition and immune suppression,^15^ and observational studies have shown that it is associated with poor prognosis in patients with ST elevation MI.^16^ However, studies investigating total lymphocyte counts and cardiovascular diseases in the general population did not find significant associations^10^ ^11^ ^17–26^ (see online supplementary table S2).

Previous studies investigating associations of eosinophil and lymphocyte counts with future risk of cardiovascular disease were limited by small size (no more than 2000 events), and they investigated only a narrow range of cardiovascular end points. It is not known how eosinophil and lymphocyte counts are associated with stroke, heart failure and peripheral arterial disease (PAD), or whether short-term and long-term associations differ. These cell counts are included in the complete blood count (full blood count), one of the most commonly performed blood tests in medical practice. Thus, large databases of routinely collected laboratory results and clinical information can provide a way of investigating the epidemiology of eosinophil and lymphocyte counts, and their association with cardiovascular diseases, at scale. Our new study harnessed the large CALIBER (CArdiovascular disease research using LInked Bespoke studies and Electronic health Records) linked electronic health record database^27^ (over 700 000 individuals and 50 000 cardiovascular events) to examine associations of eosinophil and lymphocyte counts with a diverse range of initial presentations of cardiovascular disease.

## Methods

### Study population

The study population was drawn from the CALIBER programme,^27^ which has been used for a series of studies investigating risk factors and the onset of cardiovascular diseases.^28–32^ CALIBER links four sources of electronic health data in England: primary care health records (coded diagnoses, clinical measurements and prescriptions) from general practices contributing to the Clinical Practice Research Datalink^33^ (CPRD), coded hospital discharges (Hospital Episode Statistics, HES), the Myocardial Ischaemia National Audit Project^34^ (MINAP) and death registrations (see online supplementary methods for more details). The patients in CALIBER are broadly representative of the English population.^27^ The study period was January 1998 to March 2010, and individuals were eligible for inclusion when they were at least 30 years of age and had been registered for at least 1 year in a practice which met research data recording standards. The study start date (index date) for each participant was the date of the first full blood count measurement recorded in CPRD while the participant was eligible. Patients with a prior history of cardiovascular disease and women with a pregnancy record within 6 months of the start of the study were excluded. Pregnancy or any other event occurring after study entry was not used as an exclusion criterion.

Approval was granted by the Independent Scientific Advisory Committee of the Medicines and Healthcare products Regulatory Agency (protocol 12_153) and the MINAP Academic Group. This study is registered with ClinicalTrials.gov, number NCT02014610.

### Exposure

The main exposures were eosinophil and lymphocyte counts as recorded in primary care. If a patient had more than one measurement on a given day, the values were aggregated by taking the mean. We analysed eosinophil and lymphocyte counts as categorical variables in order to avoid presuming a particular shape for the association with cardiovascular diseases. There were no clinically obvious cutpoints or consistent definitions of ‘normal’ lymphocyte or eosinophil counts in the literature. In the absence of a clear rationale for choosing specific cutpoints, we chose to study quintiles; however, the number of decimal places varied between laboratories (units: cells×10^9^/L) and the absolute values of eosinophil counts were small relative to the precision of recording (see online supplementary figure S1). In order to avoid biasing the category allocation by precision, we manually adjusted the eosinophil category boundaries, so that the second decimal place was 5, thus ensuring that any values recorded to two decimal places would end up in the same category if they were recorded to only one decimal place. We derived quintile-based categories for lymphocyte counts by a similar method. All category intervals were closed at the lower bound and open at the upper bound, that is, ‘0.05 to 0.15’ includes 0.05 but not 0.15.

Eosinophil and lymphocyte counts can be affected by many factors such as infections, autoimmune diseases, medication and haematological conditions. We sought to differentiate between a patient's long-term ‘stable’ leucocyte profile and results obtained when the patient had an ‘acute’ condition which may alter leucocyte counts. We used other information in the electronic health record to assess whether the patient was clinically ‘acute’ or ‘stable’ at the time of the blood test, adapting a set of criteria proposed by the eMERGE consortium^35^ (electronic Medical Records and Genomics) for studying genetic determinants of the stable leucocyte counts: in hospital on the date of blood test, vaccination in the previous 7 days, anaemia diagnosis within the previous 30 days, symptoms or diagnosis of infection within the previous 30 days, prior diagnosis of myelodysplastic syndrome, prior diagnosis of haemoglobinopathy, cancer chemotherapy or granulocyte colony stimulating factor (G-CSF) within 6 months before index date, or the use of drugs affecting the immune system such as methotrexate or steroids within the previous 3 months. Patients with HIV, splenectomy or on dialysis were excluded from this study, as their leucocyte counts may be difficult to interpret.

In secondary analyses, we explored associations between onset of cardiovascular diseases and the mean of the first two ‘stable’ measurements of eosinophil count taken since the start of eligibility. We also carried out a sensitivity analysis excluding patients with a history of prior loop diuretic use, as they might have symptoms of heart failure without a formal diagnosis.

### Covariates

We extracted demographic variables, cardiovascular risk factors, comorbidities, acute conditions and prescriptions around the time of the blood test from CPRD. For continuous covariates, we used the most recent value in the year prior or up to 1 day after the full blood count measurement. We also extracted the first measurement after this time window and the most recent measurement before the time window, along with the timing of these measurements relative to the index date, to use as auxiliary variables for multiple imputation. We extracted hospitalisation records and comorbidities additionally from HES.

### Follow-up and cardiovascular end points

Patients were followed up while registered at the practice until the occurrence of an initial presentation of cardiovascular disease, death or transfer out of the practice.

The primary end point was the first record of one of the following cardiovascular presentations in any of the data sources: coronary artery disease (categorised as stable angina, unstable angina, non-fatal MI, unheralded coronary death or unspecified), heart failure, transient ischaemic attack, stroke (categorised as ischaemic, haemorrhagic or unspecified), subarachnoid haemorrhage, PAD, abdominal aortic aneurysm and a composite of ventricular arrhythmia, implantable cardioverter defibrillator, cardiac arrest or sudden cardiac death. Any events occurring after the first cardiovascular presentation were ignored. End point definitions are described in online supplementary methods, and phenotyping algorithms are available on the CALIBER portal (http://www.caliberresearch.org/portal/).

### Statistical analysis

We generated cumulative incidence curves by category of eosinophil count under a competing risks framework. We used the Cox proportional hazards model to generate cause-specific hazards for association of eosinophil or lymphocyte count category with the different cardiovascular end points. We designated the middle category as the reference because we did not predetermine the direction or shape of association we were looking for. HRs were adjusted for age (linear and quadratic), sex, age/sex interaction, index of multiple deprivation, ethnicity, smoking status, diabetes, body mass index, systolic blood pressure, estimated glomerular filtration rate, high-density lipoprotein cholesterol, total cholesterol, atrial fibrillation, inflammatory conditions (autoimmune conditions, chronic obstructive pulmonary disease or inflammatory bowel disease), cancer, statin use, blood pressure medication and acute conditions at the time of blood testing as listed previously. The baseline hazard was stratified by practice and sex. We handled missing baseline covariate data by multivariate imputation using chained equations using Random Forest multiple imputation models,^36^ as described more fully in the online supplementary methods.

In supporting analyses, we examined linear associations with eosinophil count as a continuous variable, which would have greater power to detect linear associations and would enable such associations to be communicated in a more concise way. We plotted Schoenfeld residuals to assess the proportional hazards assumption, and split the follow-up time if HRs changed over time. We investigated interactions with age and sex, and carried out a sensitivity analysis excluding patients with probable symptomatic heart failure (as evidenced by loop diuretic prescription in the absence of a formal diagnosis of heart failure). We also investigated the variability of stable eosinophil and lymphocyte counts. Statistical analyses were performed using R V.2.14.1 for Linux.

## Results

We included 621 052 patients with differential leucocyte counts while clinically ‘stable’ and 154 179 patients with differential leucocyte counts performed during acute illness or treatment (see online supplementary figure S2 and table S3). We observed 55 004 initial presentations of cardiovascular disease over median 3.8 (IQR 1.7–6.0) years follow-up; 9711 occurred within the first 6 months. Of the remaining patients, 32 591 died of non-cardiovascular causes, 99 622 left the practice during the study period and were censored, and 588 014 were still registered with the practice at the end of the study period.

### Baseline characteristics

Lower eosinophil and lymphocyte counts were not associated with major cardiovascular risk factors. Low eosinophil count was associated with female gender, black ethnicity and cancer, whereas higher eosinophil count was associated with male sex, deprivation, South Asian ethnicity, higher body mass index and atopy (table 1, see online supplementary table S4). Low lymphocyte count was associated with cancer, in common with eosinophil counts, but the association with ethnicity was different: people of black ethnicity tended to have higher lymphocyte counts (table 1, see online supplementary table S5). Eosinophil and lymphocyte counts in the lowest categories were more likely to be taken under acute conditions (see online supplementary tables S6 and S7).

**Table 1:** Characteristics of patients with low eosinophil or low lymphocyte counts

Patient group	All patients	Low eosinophils (<0.05×10^9^/L)	Low lymphocytes (<1.45×10^9^/L)
N patients	775 231	44 112	133 220
Women, n (%)	461 956 (59.6%)	29 990 (68.0%)	80 119 (60.1%)
Age, median (IQR)	52.4 (41.4–64.6)	52 (39.8–66.8)	55.8 (43.4–70.4)
Most deprived quintile, n (%)	132 971 (17.2%)	6931 (15.8%)	18 982 (14.3%)
Ethnicity, n (%)			
White	445 034 (92.8%)	25 511 (91.7%)	80 558 (95.3%)
South Asian	13 450 (2.8%)	552 (2.0%)	1134 (1.3%)
Black	9974 (2.1%)	1051 (3.8%)	1214 (1.4%)
Other	11 320 (2.4%)	714 (2.6%)	1598 (1.9%)
Missing	295 453 (38.1%)	16 284 (36.9%)	48 716 (36.6%)
Smoking status, n (%)			
Never	386 702 (52.6%)	25 034 (61.0%)	76 478 (61.6%)
Ex	177 199 (24.1%)	8840 (21.5%)	30 682 (24.7%)
Current	171 021 (23.3%)	7158 (17.4%)	16 925 (13.6%)
Missing	40 309 (5.2%)	3080 (7.0%)	9135 (6.9%)
Most recent value within 1 year prior to index date, median (IQR)			
Systolic blood pressure	136 (121–150)	133 (120–148)	136 (120–150)
Body mass index	27.0 (23.8–31.1)	25.0 (22.0–28.5)	25.5 (22.5–29.0)
Total cholesterol	5.5 (4.8–6.2)	5.4 (4.6–6.1)	5.3 (4.6–6.1)
HDL cholesterol	1.4 (1.1–1.7)	1.5 (1.2–1.8)	1.5 (1.2–1.8)
eGFR	81.9 (68.8–95.1)	81.4 (67.6–95.4)	78.9 (65.1–92.1)
Diagnoses on or before index date, n (%)			
Atrial fibrillation	7722 (1.0%)	555 (1.3%)	2358 (1.8%)
Cancer	47 521 (6.1%)	3895 (8.8%)	13 109 (9.8%)
Diabetes	37 127 (4.8%)	1502 (3.4%)	5807 (4.4%)
Asthma or atopy	212 900 (27.5%)	9783 (22.2%)	36 750 (27.6%)
COPD	15 021 (1.9%)	863 (2.0%)	3250 (2.4%)
Connective tissue disease	21 859 (2.8%)	1680 (3.8%)	5629 (4.2%)
Inflammatory bowel disease	8646 (1.1%)	704 (1.6%)	2337 (1.8%)

COPD, chronic obstructive pulmonary disease; eGFR, estimated glomerular filtration rate; HDL, high-density lipoprotein.

### Cumulative incidence of cardiovascular disease by eosinophil and lymphocyte count

**Figure 1:**
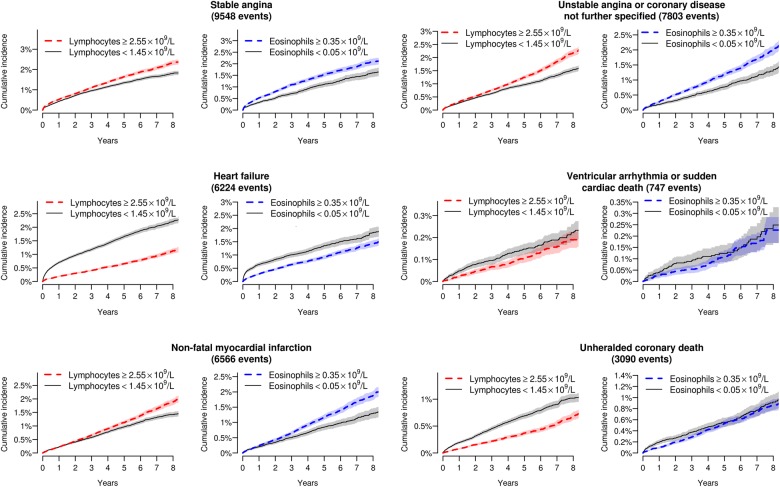
Cumulative incidence curves for different initial presentations of cardiac disease by category of eosinophil and lymphocyte count. Shading denotes 95% CIs.

**Figure 2:**
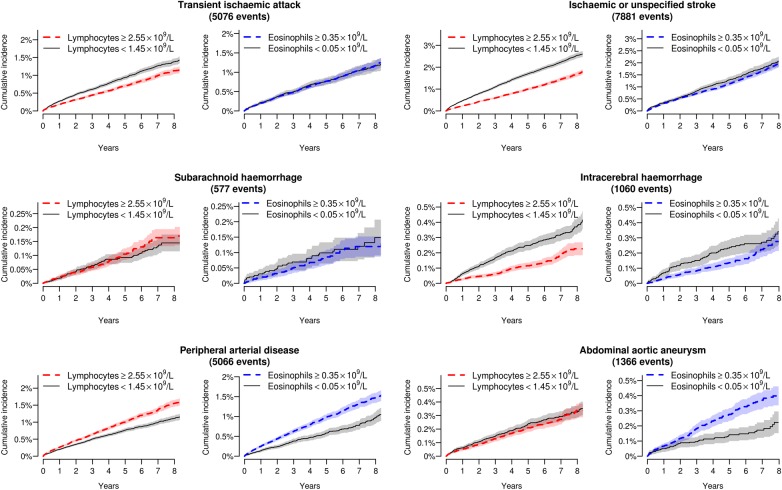
Cumulative incidence curves for non-cardiac initial presentations of cardiovascular disease by category of eosinophil and lymphocyte count. Shading denotes 95% CIs.

Crude cumulative incidence curves showed that individuals with low eosinophil or lymphocyte counts had a greater incidence of heart failure as the initial presentation of cardiovascular disease, at least over the first few months (figure 1), but there was no apparent association with non-cardiac presentations of cardiovascular disease (figure 2).

### HRs for association of eosinophil and lymphocyte counts with initial presentation of cardiovascular disease

In multiply adjusted analyses, there were associations of low eosinophil counts (<0.05 compared with the middle category, 0.15–0.25×10^9^/L) with incident heart failure (adjusted HR 1.39; 95% CI 1.26 to 1.54), unheralded coronary death (HR 1.29, 95% CI 1.12 to 1.50) and ventricular arrhythmia or sudden cardiac death (HR 1.49, 95% CI 1.12 to 2.00). However, there were no such associations with stable angina (HR 0.93, 95% CI 0.84 to 1.03), non-fatal MI (HR 1.00, 95% CI 0.89 to 1.12) or ischaemic stroke (HR 1.02, 95% CI 0.88 to 1.19; see online supplementary figure S3). Low lymphocyte counts (<1.45 compared with 1.85–2.15×10^9^/L) also showed associations with heart failure (HR 1.61, 95% CI 1.48 to 1.74) and unheralded coronary death (HR 1.27, 95% CI 1.13 to 1.42; see online supplementary figure S4). Low eosinophil counts (<0.05 compared with 0.15–0.25×10^9^/L) were associated with significantly increased risk of non-cardiovascular death (HR 1.79, 95% CI 1.72 to 1.86), as were low lymphocyte counts (<1.45 compared with 1.85–2.15×10^9^/L; HR 1.65, 95% CI 1.59 to 1.71), with no clear preponderance for a particular cause of death (see online supplementary tables S8 and S9). Mutual adjustment of lymphocyte and eosinophil counts made very little difference to the HRs (see online supplementary figures S5 and S6).

In models adjusted only for age and sex, the lowest eosinophil category (<0.05 compared with 0.15–0.25×10^9^/L) was associated with reduced hazard of stable angina (HR 0.83, 95% CI 0.75 to 0.92) and PAD (HR 0.81, 95% CI 0.70 to 0.92; see online supplementary figure S7); there was a similar trend of lower risk with lower eosinophil count for non-fatal MI. These associations were not present with multiple adjustment (see online supplementary figure S3).

### Supporting analyses

**Figure 3:**
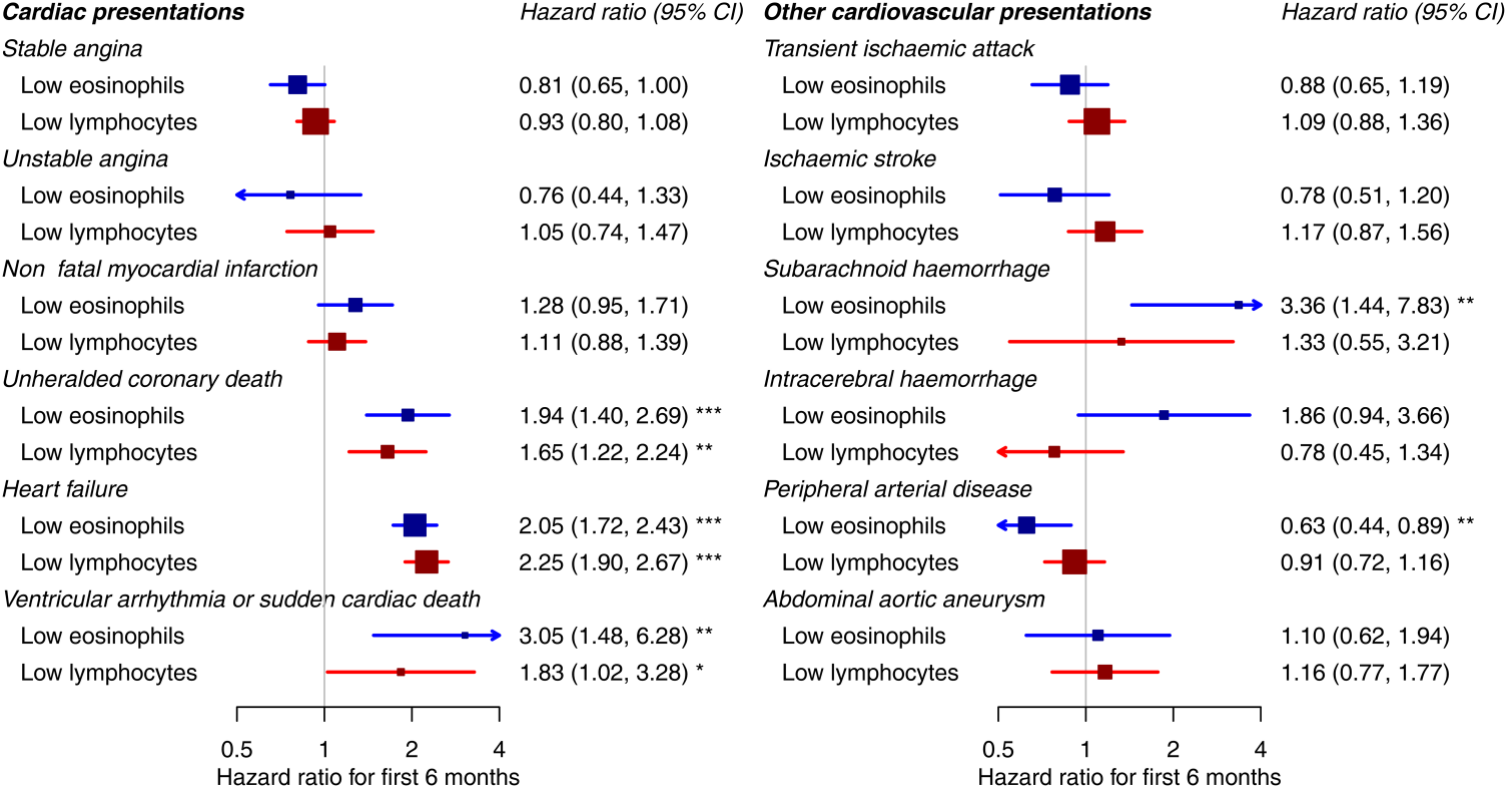
Association of low eosinophil and lymphocyte counts with different initial presentations of cardiovascular disease over the first 6 months ‘Low eosinophils’ are the lowest category (<0.05) compared with the middle category (0.15−0.25). ‘Low lymphocytes’ are the lowest category (<1.45) compared with the middle category (1.85−2.15). HRs are adjusted for age, sex, deprivation, ethnicity, smoking, diabetes, systolic blood pressure, body mass index, total cholesterol, HDL cholesterol, eGFR, atrial fibrillation, autoimmune conditions, inflammatory bowel disease, COPD, cancer, statin use, blood pressure medication and acute conditions at the time of blood testing. COPD, chronic obstructive pulmonary disease; eGFR, estimated glomerular filtration rate; HDL, high-density lipoprotein.

We found that associations with low eosinophil counts were stronger in the first 6 months and weak or null thereafter (figure 3, see online supplementary figure S8). In the first 6 months, the HR associating low eosinophil counts (<0.05 compared with 0.15–0.25×10^9^/L) with incident heart failure was 2.05 (95% CI 1.72 to 2.43). The corresponding HR for unheralded coronary death was 1.94 (95% CI 1.40 to 2.69), and for ventricular arrhythmia or sudden cardiac death 3.05 (95% CI 1.48 to 6.28), as shown in figure 3. Similar associations were shown for low lymphocyte counts (figure 3, see online supplementary figure S9).

Associations by category of eosinophil and lymphocyte counts with cardiovascular diseases were similar between acute and stable patient states (see online supplementary figures S10 and S11). Considering linear associations with eosinophil count, we found some evidence that higher stable eosinophil counts were weakly associated with increased risk of non-fatal MI (HR per 0.1×10^9^/L higher eosinophil count was 1.02, 95% CI 1.01 to 1.04, p=0.0088) and PAD (HR 1.03, 95% CI 1.01 to 1.04, p=0.003; see online supplementary figure S12). These associations were weak relative to the distribution of eosinophil counts in our population (SD 0.16×10^9^/L). When we split the follow-up time at 6 months, we found that higher eosinophil counts were associated with slightly increased risk of non-fatal MI or PAD from 6 months onwards (see online supplementary figure S13). There were no significant interactions with age or sex, and a sensitivity analysis excluding patients on loop diuretics made little difference to the results.

There was considerable variability but minimal trend over time between repeat measurements of stable eosinophil counts; the SD of differences between two consecutive measurements was 0.14×10^9^/L and the correlation coefficient was 0.597 (see online supplementary figure S14). For stable lymphocyte counts the SD of differences between two consecutive measurements was 0.59×10^9^/L and the correlation coefficient was 0.706 (see online supplementary figure S15).

## Discussion

### Summary of main findings

In a general population without pre-existing cardiovascular disease, we found that low eosinophil and lymphocyte counts were associated with increased short-term incidence of heart failure, unheralded coronary death and ventricular arrhythmia/sudden cardiac death as the initial presentation of cardiovascular disease, and of non-cardiovascular death. Previous studies have suggested such associations among patients with pre-existing heart failure,^2–5^ but our study is the first to demonstrate them in a healthy population. We also demonstrated lack of association with other cardiovascular diseases such as angina, non-fatal MI and stroke.

### Possible explanations

Eosinophils and lymphocytes are two different classes of immune cells with very different functions, so it seems remarkable that they have a similar pattern of association with specific cardiovascular diseases. As this is an observational study, residual confounding cannot be excluded, but low eosinophil and low lymphocyte counts were not associated with major cardiovascular disease risk factors (table 1, see online supplementary tables S4 and S5) and our associations were robust to a range of multivariable adjustments. Reverse causation (eg, patients developing low eosinophil count as a consequence of undiagnosed heart failure) is also an unlikely explanation for our findings, because we obtained consistent results in a sensitivity analysis excluding patients on loop diuretics. One explanation for our findings is that low eosinophil and lymphocyte counts may be markers of frailty, malnutrition^15^ or immune suppression,^37^ which increase the risk of specific cardiovascular diseases and non-cardiovascular death. Another potential explanation is that high levels of endogenous mineralocorticoids increase the risk of heart failure via sodium retention, and also suppress eosinophil and lymphocyte counts (adrenal insufficiency may be associated with eosinophilia^38^ and lymphocytosis^39^). A third explanation involves interleukin-5 (IL-5), which mediates the release of eosinophils into the bloodstream^40^ and is involved in differentiation of B lymphocytes to immunoglobulin-secreting cells.^41^ Variants in the IL-5 gene are associated with coronary artery disease,^42^ and in mouse studies macrophage-specific overexpression of IL-5 led to increased secretion of IgM antibodies that inhibit uptake of oxidised low-density lipoprotein, with consequent inhibition of atherosclerosis.^43^ In cross-sectional studies, blood levels of IL-5 were strongly associated with eosinophil counts^44^ and decreased subclinical atherosclerosis,^45^ and cohort studies among critical care patients found that higher IL-5 was associated with better outcomes.^46^ We are not aware of any longitudinal cohort studies among the general population which have investigated the association of IL-5 and cardiovascular events; we hypothesise that such an association exists.

### Association of eosinophil counts with coronary disease

In contrast to heart failure and unheralded coronary death, we found a weak positive association of increasing eosinophil count with increasing risk of MI. Previous studies reported a much stronger association;^10^ ^11^ this may be because we adjusted more completely for other cardiovascular risk factors (higher eosinophil count was associated with higher BMI, smoking, diabetes and deprivation; see table 1). Genome wide association studies found that a non-synonymous single nucleotide polymorphism rs3184504[T] in SH2B3 is associated with higher eosinophil count and MI;^47^ given that our results did not show a strong association of eosinophil count with MI, it seems likely that the causal pathway from rs3184504[T] is not mediated by eosinophils.

### Therapeutic implications

Anti-IL5 therapy is being investigated as a therapy for asthma, which is characterised by an overactive immune response and high eosinophil counts.^48^ If IL-5 truly has an atheroprotective effect, patients with asthma treated with anti-IL-5 may potentially be at increased cardiovascular risk, and this should be monitored in clinical trials and postmarketing surveillance. More broadly, this study suggests that eosinophil and lymphocyte counts should be interpreted in a more nuanced way in clinical practice. Currently they inform clinical management only if the results are outside the ‘normal’ (reference) range. Clinical reference ranges for eosinophil and lymphocyte counts are based on the distribution of cell counts among the population, and are not standardised between laboratories; in our university hospital, the reference ranges are 0–0.40×10^9^/L for eosinophils and 1.20–3.65×10^9^/L for lymphocytes. Thus, low eosinophil counts are considered ‘normal’ even though they are associated with increased risk of heart failure and death.

### Research implications

We recommend that these findings are replicated in bespoke cohort studies, with blood counts performed under standardised conditions and concurrent measurement of IL-5. It would also be of interest to study lymphocyte subsets, as different types of lymphocytes are affected by different regulatory mechanisms. Associations of lymphocyte subsets with cardiovascular diseases have only been investigated in small studies with only a few hundred patients.^49^ Findings from highly phenotyped, bespoke cohorts will complement the ‘real-world’ epidemiological findings of this study and yield further mechanistic insights.

### Limitations

Although our study has strengths—large size, population base, extensive adjustment for potential confounders—it also has important limitations. One of the main limitations is the selection bias, as participants were only included if they had a full blood count performed in usual clinical care, and the indications for this test could vary widely. However, we investigated conditions and medication that may acutely affect leucocyte counts, and obtained consistent results in a range of sensitivity analyses, so we consider that our results are generalisable. Another limitation is that the measurement of lymphocyte and eosinophil counts was undertaken by a large number of different laboratories without study-wide protocols; this may have led to underestimating the association because of random error (regression dilution bias).

As our study was based on electronic health records, some values of baseline variables were missing for some patients, but we obtained similar results by imputing missing data using two different methods. The ascertainment of end points was via routinely coded clinical data, without end point adjudication;^50^ however, any failures in end point recording are likely to be non-differential in relation to the eosinophil count. Finally, as with any observational study, the results cannot be taken to imply causation because there is the possibility of residual confounding. We feel this is unlikely because of the strength of the associations observed and the wide range of potential confounders included in our models.

## Conclusions

Low eosinophil and lymphocyte counts were strongly associated with increased short-term incidence of heart failure and coronary death in a healthy population. This may have implications for monitoring the cardiovascular safety of anti-IL-5 therapy for asthma, and for the interpretation of these tests in clinical practice.
