# Supplementary material for: Low eosinophil and low lymphocyte counts and the incidence of 12 cardiovascular diseases: a CALIBER cohort study
Source: Open Heart. 2016 Sep 5;3(2):e000477. doi: 10.1136/openhrt-2016-000477 (PMC5013342; doi:10.1136/openhrt-2016-000477)
Supplement: Supplementary data [file openhrt-2016-000477supp.pdf]

Low eosinophil and low lymphocyte counts and the incidence of twelve cardiovascular diseases: a CALIBER cohort study

Supplementary Material

July 28, 2016

Contents

Supplementary Methods 3

Study data sources . . . 3

Endpoint definitions . . . 3

Survival analysis and competing risks . . . 4

Multiple imputation . . . 5

Supplementary Tables 7

Supplementary Table 1. Cohort studies with over 2000 participants investigating eosinophil counts and incidence of cardiovascular diseases . . . 7

Supplementary Table 2. Cohort studies with over 2000 participants investigating lymphocyte counts and incidence of cardiovascular diseases . . . 8

Supplementary Table 3. Characteristics of patients and prevalence of conditions defining 'acute' and 'stable' full blood counts . . . 9

Supplementary Table 4. Characteristics of patients by category of eosinophil count . . . 10

Supplementary Table 5. Characteristics of patients by quintile of lymphocyte count . . . 11

Supplementary Table 6. Prevalence of acute conditions on date of blood testing by category of eosinophil count . . . 12

Supplementary Table 7. Prevalence of acute conditions on date of blood testing by quintile of lymphocyte count . . . 13

Supplementary Table 8. Endpoints by category of eosinophil count . . . 14

Supplementary Table 9. Endpoints by quintile of lymphocyte count . . . 15

Supplementary Figures 16

Supplementary Figure 1. Histogram showing distribution of eosinophil counts and category cutpoints 16

Supplementary Figure 2. Patient flow diagram . . . 17

Supplementary Figure 3. Multivariable adjusted association of eosinophil categories with different initial presentations of cardiovascular disease . . . 18

Supplementary Figure 4. Multivariable adjusted association of lymphocyte quintiles with different initial presentations of cardiovascular disease 19
Supplementary Figure 5. Association of eosinophil categories with different initial presentations of cardiovascular disease, adjusted for lymphocyte count 20
Supplementary Figure 6. Association of lymphocyte quintiles with different initial presentations of cardiovascular disease, adjusted for eosinophil count 21
Supplementary Figure 7. Age and sex adjusted association of eosinophil categories with different initial presentations of cardiovascular diseases 22
Supplementary Figure 8. Multivariable adjusted association of eosinophil categories with different initial presentations of cardiovascular disease, by time after measurement 23
Supplementary Figure 9. Multivariable adjusted association of lymphocyte quintiles with different initial presentations of cardiovascular disease, by time after measurement 24
Supplementary Figure 10. Association of eosinophil categories with different initial presentations of cardiovascular disease, by patient state at the time of blood sampling 25
Supplementary Figure 11. Association of lymphocyte quintiles with different initial presentations of cardiovascular disease, by patient state at the time of blood sampling 26
Supplementary Figure 12. Linear association of eosinophil counts with different initial presentations of cardiovascular disease, by patient state at the time of blood sampling 27
Supplementary Figure 13. Linear association of eosinophil counts with different initial presentations of cardiovascular disease, by time since blood sampling 28
Supplementary Figure 14. Binned scatterplot showing difference between two consecutive eosinophil counts taken when a patient was clinically 'stable' 29
Supplementary Figure 15. Binned scatterplot showing difference between two consecutive lymphocyte counts taken when a patient was clinically 'stable' 30

Supplementary References **31**

Supplementary Methods

Study data sources

The CALIBER research platform (Cardiovascular disease research using Linked Bespoke studies and Electronic Records) [1] contains linked electronic health records from four data sources in England:

1. Primary care data from 225 general practices in the Clinical Practice Research Datalink (CPRD) [2]. CPRD provides primary care data on demographics, ethnicity, health behaviours, diagnoses, investigations, procedures and prescriptions. Diagnoses are coded using the Read Clinical Terminology. (Read Terms are a major component of the SNOMED-CT terminology).

2. Hospital Episodes Statistics (HES), containing details of hospital admissions (http://www.hscic.gov.uk/hes). Diagnoses are coded using the International Statistical Classification of Diseases and Health Related Problems, 10th revision (ICD-10); interventions are coded using the Office of the Population Censuses and Surveys Classification of Interventions and Procedures (OPCS). Ethnicity as recorded during hospital attendances is also included in HES.

3. Details of acute coronary syndromes from the Myocardial Ischaemia National Audit Project registry (MINAP) [3].

4. Date and ICD-10 coded cause of death from the Office for National Statistics (ONS) death registry. The index of multiple deprivation according to the patient's area of residence was also obtained from ONS.

The linkage was carried out in October 2010 by a trusted third party, using a deterministic match between NHS number, date of birth, and sex. Overall, 96% of patients with a valid NHS number were successfully matched [4].

Endpoint definitions

Cardiovascular phenotype definitions based on the CALIBER data sources are curated on the CALIBER data portal (www.caliberresearch.org/portal). The endpoint was the first occurrence of one of the following cardiovascular presentations. If more than one endpoint was recorded on the same date, the lowest numbered endpoint was allocated.

1. Ventricular arrhythmia or sudden cardiac death was a composite of ventricular arrhythmias, implantable cardioverter defibrillator, and sudden cardiac death. It was defined using diagnoses and procedure codes in primary care, secondary care and death certificates.

2. Heart failure was defined by coded diagnoses in primary care, secondary care and death certificates.

3. Unheralded coronary death was death with the primary cause certified as coronary heart disease, and no prior history of cardiovascular disease. Patients with myocardial infarction who died on the day of their infarct were considered to have unheralded coronary death.

4. Non-fatal myocardial infarction was defined as a disease registry diagnosis of an acute coronary syndrome with elevated troponin, or a primary or secondary care diagnosis of myocardial infarction.

5. Unstable angina was defined as a primary or secondary care diagnosis of unstable angina, or an acute coronary syndrome without myocardial infarction recorded in the disease registry.

6. Stable angina was defined by a coded diagnosis in primary or secondary care of ischaemic chest pain or stable angina, a positive myocardial ischaemia test, two or more prescriptions of antianginal medication, or coronary revascularisation.

7. Coronary disease not further specified is a non-specific diagnosis of ischaemic or coronary heart disease in primary or secondary care that does not fall into one of the more specific categories. It was not included among the 12 diseases in the main displays of hazard ratios, but for cumulative incidence calculations it was combined with unstable angina.

8. Abdominal aortic aneurysm was defined by coded diagnoses and procedures in primary care, secondary care and death certificates.

9. Peripheral arterial disease includes intermittent claudication, limb ischaemia or gangrene due to atherosclerotic disease in the arteries of the legs. It was defined by coded diagnoses and procedures in primary care, secondary care and death certificates.

10. Subarachnoid haemorrhage was defined by coded diagnoses in primary care, secondary care and death certificates.

11. Intracerebral haemorrhage was defined by coded diagnoses in primary care, secondary care and death certificates.

12. Ischaemic stroke was defined using coded diagnoses in primary care, secondary care and death certificates. Patients with a procedure code for carotid endarterectomy within 90 days of a stroke of unspecified type were considered to have ischaemic stroke.

13. Stroke not further specified is a diagnosis of stroke which does not state it is ischaemic or haemorrhagic. This clinical event was not included among the 12 diseases in the main displays of hazard ratios, but for cumulative incidence calculations it was combined with ischaemic stroke.

14. Transient ischaemic attack was defined by coded diagnoses in primary or secondary care.

Survival analysis and competing risks

We carried out survival analysis to describe and model the first occurrence of any cardiovascular disease. A patient's follow-up ended when they experienced one of the cardiovascular endpoints or when they were censored. Subsequent events (e.g. myocardial infarction occurring after stable angina) were not analysed.

To describe the incidence of each initial presentation over time we constructed cumulative incidence curves, taking into account the other possible initial presentations as competing events. Normal-based confidence intervals were constructed based on Greenwood's variance formula, as implemented in the R prodlim package.

For multivariable modelling we considered using Cox models (for modelling cause-specific hazards) or the Fine and Gray model (to compare cumulative incidence curves by modelling subdistribution hazards). As the

aim of this study was observational epidemiology -– to explore associations rather than predict risk — we considered cause specific hazard ratios to be appropriate quantities to estimate. They should be interpreted together with cumulative incidence curves but cannot be used to predict cumulative incidence. We also found the Fine and Gray model to be more computationally intensive, and it would require significant software engineering or computing time to apply it to the large dataset used in these analyses. We used follow-up time as the timescale for the Cox models in order to investigate how the strength of association between a leukocyte count measurement and outcome varies depending on time since measurement, and determine whether it is more useful for short / medium or long term prediction.

We assessed the proportional hazards assumption by plotting scaled Schoenfeld residuals against time. We found evidence of non-proportional hazards for many of the endpoints, with a stronger association in the first few months, so we split the follow-up time at 6 months in order to demonstrate the change in the strength of association over time.

Multiple imputation

We used two methods of multiple imputation for imputing missing data: Random Forest and normal-based MICE. For the primary analysis we used Random Forest multiple imputation, as implemented in the CALIBERrfimpute package, as it can account for interactions between predictor variables without requiring them to be explicitly specified in the imputation models [5]. Categorical variables were imputed using the 'rfcat' function, in which each imputed value is the prediction from a randomly chosen tree. Continuous variables were imputed using the 'rfcont' function, in which imputed values are randomly drawn from normal distributions centred on imputed means estimated using Random Forest.

The Random Forest algorithm has theoretical advantages but it is new, so we also applied the established method of normal-based MICE imputation. We included the following variables in imputation models:

- Complete blood count parameters: total white cell count, neutrophil count, lymphocyte count, eosinophil count, basophil count, monocyte count, haemoglobin concentration, platelet count. For normal-based imputation, we included leukocyte subtype counts as categorical variables (quintiles) as they would be used in the analysis, in order to avoid imposing linearity in the imputation models.
- Cardiovascular risk factors including those in the substantive models: age, age squared, sex, body mass index, blood pressure, diabetes, smoking, total cholesterol, HDL cholesterol, triglycerides, eGFR, index of multiple deprivation
- Event indicator
- Type of event
- Time as the marginal Nelson-Aalen cumulative hazard
- Use of statins or blood pressure lowering medication in the year before study entry
- Conditions potentially affecting blood counts, based on eMERGE criteria [6, 7]: haemoglobinopathy, prior diagnosis of myelodysplasia, anaemia diagnosis within 30 days prior, anaemia diagnosis in following 30 days, prior diagnosis of cancer, cancer diagnosis in following 2 years, chemotherapy within 6 months prior, chemotherapy in following 3 months, renal dialysis prior, HIV diagnosis prior, steroid prescription within 3 months prior, methotrexate prescription within 3 months prior, prescription for another drug affecting the immune system within 30 days prior, infection diagnosis within 30 days prior,

infection diagnosis within 30 days after, infective symptoms within 30 days prior, infective symptoms within 30 days after, splenectomy prior, immunisation within 1-7 days prior

- Prior record of other comorbidities: atrial fibrillation, chronic obstructive pulmonary disease, inflammatory bowel disease, atopy, asthma, cancer, systemic autoimmune conditions

For imputing continuous measurements using Random Forest, we additionally used the last measurement before the 1-year time window before study entry, and the first measurement after study entry, along with the timing of these measurements relative to the study start date, as auxiliary variables for the imputation of that variable.

We log transformed skewed variables (HDL cholesterol, eGFR, total cholesterol and triglycerides) before imputation because both rfcont and normal-based multiple imputation functions assume a normal distribution of residuals. We exponentiated the imputed values and truncated the upper end of the distribution to the maximum value in the original data (this affected only a small number of observations, but the values were very large and would affect any normal-based linear modelling). We split the dataset was split by gender and geographical region for multiple imputation, with general practice included as a categorical variable. We generated imputations in parallel on the CALIBER high performance computing cluster. We generated 20 imputations, each drawn from 20 iterations of MICE. We reviewed plots of chain means and variances of imputed variables to verify that 20 was a sufficient number of iterations. We combined the results of Cox models using Rubin's rules [8]. We verified that the ratio of between-imputation variance to total variance ('fraction of missing information') for each parameter of interest was less than 20%, implying that 20 imputations were sufficient.

Supplementary Tables

Supplementary Table 1. Cohort studies with over 2000 participants investigating eosinophil counts and incidence of cardiovascular diseases

Author, year	Study population	N patients	Lowest eosinophil category investigated	Endpoint	N events	Adjusted measure of association
Prentice, 1982 [9]	Hiroshima and Nagasaki	12 858	$< 0.12 \times 10^9$/L	Coronary heart disease	153	RR 1.78 (P = 0.01) for eosinophils$\geq 0.3 \times 10^9$/L vs $< 0.12 \times 10^9$/L
Sweetnam, 1997 [10]	Caerphilly (men only)	2163	Bottom quintile	Coronary death or myocardial infarction	143	HR 2.15 for top vs bottom quintile (P for trend 0.05)
Olivares, 1993 [11]	Male civil servants and railway employees (France)	3659	None (linear only)	Coronary heart disease	46	RR 1.03 (95% CI 0.87, 1.21) per 10^8/L higher
Current study: CALIBER	Population-based, 225 English general practices	775 231	$< 0.05 \times 10^9$/L	12 initial cardiovascular presentations	55 004	HR over first 6 months for eosinophils <0.05 vs 0.15–0.25 $\times 10^9$/L: heart failure 2.05 (95% CI 1.72, 2.43), unheralded coronary death 1.94 (95% CI 1.40, 2.69)

CI, confidence interval; HR, hazard ratio; RR, relative risk.

Supplementary Table 2. Cohort studies with over 2000 participants investigating lymphocyte counts and incidence of cardiovascular diseases

Author, year	Study population	N patients	Endpoint	N events	Adjusted measure of association
Adamsson Eryd, 2012 [12]	Malmo Diet and Cancer Study	27 085	Coronary death or myocardial infarction	1965	HR 1.05 (95% CI 1.00, 1.11) per SD (0.88 $\times 10^9$/L) higher
Bekwelem, 2011 [13]	ARIC	14 485	Heart failure	1647	HR 0.86 for top vs bottom quintile
Zia, 2012 [14]	Malmo Diet and Cancer Study	26 927	Cerebral infarction	1314	HR 1.2 (95% CI 0.98, 1.4) for top vs bottom quartile
Pfister, 2012 [15]	EPIC-Norfolk	16 011	Heart failure	935	HR (95% CI) per 10^9/L higher: men 0.97 (0.83, 1.13) women 0.92 (0.77, 1.10)
Gillum, 2005 [16]	NHEFS	4625	Coronary artery disease or coronary death	914	HR 1.05 (0.89, 1.24) for top vs bottom tertile
Wheeler, 2004 [17]	ARIC	11 337	Coronary death or myocardial infarction	531	RR 1.2 for top vs bottom tertile (read off graph in meta-analysis)
Prentice, 1982 [18]	Hiroshima and Nagasaki	13 040	Cerebral infarction<br>Intracerebral haemorrhage	336<br>73	HR 1.01 per 10^9/L higher (P = 0.89)<br>HR 0.73 per 10^9/L higher (P = 0.07)
Karino, 2015 [19]	Honolulu Heart Program	2879	acute coronary syndrome or coronary death	279	HR 1.14 (95% 0.80, 1.62) for top vs bottom quartile (P for trend 0.94)
Shah, 2014 [20]	National Health and Nutrition Examination Survey-III	7250	Nonfatal myocardial infarction, fatal coronary disease	231	HR 1.33 (95% CI 0.81, 2.18) for > 1.5 vs $\leq 1.5 \times 10^9$/L
Prentice, 1982 [9]	Hiroshima and Nagasaki	12 858	Angina, myocardial infarction or coronary death	154	HR 1.05 per 10^9/L higher (P = 0.62)
Sweetnam, 1997 [10]	Caerphilly (men only)	2163	Coronary death or myocardial infarction	143	HR 1.08 for top vs bottom quintile (P for trend 0.21)
Olivares, 1993 [11]	Paris Prospective Study II	3659	Coronary heart disease	46	RR 1.06 (95% CI 0.73, 1.55) per 10^9/L higher
Current study: CALIBER	Population-based, 225 English general practices	775 231	12 initial cardiovascular presentations	55 004	HR over first 6 months for lymphocytes <1.45 vs 1.85–2.15 $\times 10^9$/L: heart failure 2.25 (95% CI 1.90, 2.67), unheralded coronary death 1.65 (95% CI 1.22, 2.24)

CI, confidence interval; HR, hazard ratio; RR, relative risk.

Supplementary Table 3. Characteristics of patients and prevalence of conditions defining 'acute' and 'stable' full blood counts

		Full blood count (FBC) recorded while eligible for study		
	**No FBC**	**Patients excluded**	**'Acute' FBC**	**'Stable' FBC**
N patients	1 034 863	1630	154 179	621 052
Women, n (%)	455 290 (44.0%)	780 (47.9%)	94 383 (61.2%)	367 573 (59.2%)
Age, median (IQR)	N/A	53.1 (42.7–65.0)	56.2 (43.1–68.9)	51.6 (41.1–63.4)
Most deprived quintile, n (%)	220 256 (21.4%)	328 (20.2%)	27 196 (17.7%)	105 775 (17.1%)
Duration of registration before index date (years), median (IQR)	N/A	11.3 (4.09–19.1)	11.1 (4.21–20.3)	10.9 (4.22–19.6)
Eosinophil count $\times 10^9$/L, median (IQR)	N/A	0.2 (0.1–0.3)	0.17 (0.1–0.26)	0.18 (0.1–0.24)
Ethnicity, n (%):				
White	389 590 (87.1%)	1086 (91.3%)	96 591 (92.7%)	348 443 (92.8%)
South Asian	14 697 (3.3%)	12 (1.0%)	3015 (2.9%)	10 435 (2.8%)
Black	20 471 (4.6%)	68 (5.7%)	2301 (2.2%)	7673 (2.0%)
Other	22 511 (5.0%)	24 (2.0%)	2282 (2.2%)	9038 (2.4%)
Missing	587 594 (56.8%)	440 (27.0%)	49 990 (32.4%)	245 463 (39.5%)
Recording within 1 year before study entry:				
HDL and total cholesterol		448 (27.5%)	40 520 (26.3%)	204 507 (32.9%)
Blood pressure		1062 (65.2%)	97 069 (63.0%)	409 775 (66.0%)
eGFR		892 (54.7%)	79 971 (51.9%)	284 628 (45.8%)
Body mass index		540 (33.1%)	47 194 (30.6%)	195 144 (31.4%)
Person level exclusions (eMERGE):				
HIV before index date		156 (9.6%)	0	0
Dialysis before index date		287 (17.6%)	0	0
Splenectomy before index date		1189 (72.9%)	0	0
Conditions at the time of FBC measurement (adapted from eMERGE):				
In hospital on date of blood test		25 (1.5%)	3080 (2.0%)	0
Vaccination within previous 7 days		53 (3.3%)	8171 (5.3%)	0
Anaemia diagnosis within 30 days before		24 (1.5%)	3757 (2.4%)	0
Infection diagnosis within 30 days before		174 (10.7%)	54 251 (35.2%)	0
Infective symptoms within 30 days before		118 (7.2%)	43 178 (28.0%)	0
Prior diagnosis of myelodysplastic syndrome		7 (0.4%)	306 (0.2%)	0
Prior diagnosis of haemoglobinopathy		11 (0.7%)	2649 (1.7%)	0
Chemotherapy or G-CSF within 6 months before		19 (1.2%)	2056 (1.3%)	0
Methotrexate within 3 months before		2 (0.1%)	3077 (2.0%)	0
Steroid within 3 months before		152 (9.3%)	21 176 (13.7%)	0
Other immune drug within 3 months before		161 (9.9%)	8393 (5.4%)	0

Supplementary Table 4. Characteristics of patients by category of eosinophil count

**Category**	**1 (lowest)**	**2**	**3**	**4**	**5 (highest)**
**Eosinophils, $\times 10^9$/L**	**< 0.05**	**0.05–0.15**	**0.15–0.25**	**0.25–0.35**	**≥ 0.35**
N patients	44 112	307 668	228 639	106 092	88 720
Women, n (%)	29 990 (68.0%)	199 375 (64.8%)	132 275 (57.9%)	55 902 (52.7%)	44 414 (50.1%)
Age, median (IQR)	52 (39.8-66.8)	52.1 (41-64.3)	53 (42.1-64.6)	52.8 (42-64.5)	51.9 (41.1-64.4)
Most deprived quintile, n (%)	6931 (15.8%)	48 164 (15.7%)	40 077 (17.6%)	19 886 (18.8%)	17 913 (20.3%)
Ethnicity, n (%):					
White	25 511 (91.7%)	175 418 (93.0%)	131 908 (93.5%)	61 286 (92.8%)	50 911 (90.7%)
South Asian	552 (2.0%)	4187 (2.2%)	3774 (2.7%)	2238 (3.4%)	2699 (4.8%)
Black	1051 (3.8%)	4496 (2.4%)	2356 (1.7%)	1017 (1.5%)	1054 (1.9%)
Other	714 (2.6%)	4564 (2.4%)	3056 (2.2%)	1501 (2.3%)	1485 (2.6%)
Missing	16 284 (36.9%)	119 003 (38.7%)	87 545 (38.3%)	40 050 (37.8%)	32 571 (36.7%)
Complete blood count parameters on index date, median (IQR):					
Neutrophils, $\times 10^9$/L	3.98 (2.8-5.7)	3.7 (2.9-4.8)	3.9 (3.1-4.97)	4 (3.2-5.12)	4.2 (3.3-5.36)
Lymphocytes, $\times 10^9$/L	1.6 (1.2-2)	1.87 (1.5-2.3)	2 (1.66-2.5)	2.1 (1.71-2.6)	2.2 (1.8-2.73)
Monocytes, $\times 10^9$/L	0.42 (0.3-0.6)	0.44 (0.35-0.6)	0.5 (0.4-0.6)	0.5 (0.4-0.64)	0.53 (0.4-0.7)
Basophils, $\times 10^9$/L	0 (0-0.02)	0.02 (0-0.05)	0.03 (0-0.08)	0.04 (0-0.1)	0.05 (0-0.1)
Haemoglobin, g/dL	13.5 (12.5-14.5)	13.8 (12.9-14.8)	14 (13.1-15)	14.1 (13.1-15.1)	14.1 (13.1-15.1)
Platelets, $\times 10^9$/L	249 (206-297)	255 (217-299)	261 (223-306)	266 (227-312)	273 (233-321)
Smoking status, n (%):					
Never	25 034 (61.0%)	167 598 (57.6%)	109 365 (50.3%)	46 699 (46.2%)	38 006 (45.1%)
Ex	8840 (21.5%)	68 595 (23.6%)	53 712 (24.7%)	25 193 (24.9%)	20 859 (24.7%)
Current	7158 (17.4%)	54 997 (18.9%)	54 292 (25.0%)	29 089 (28.8%)	25 485 (30.2%)
Missing	3080 (7.0%)	16 478 (5.4%)	11 270 (4.9%)	5111 (4.8%)	4370 (4.9%)
Most recent value within one year prior to index date, median (IQR):					
Systolic blood pressure	133 (120-148)	135 (120-150)	138 (123-150)	138 (124-150)	136 (122-150)
Body mass index	25 (22-28.5)	26.5 (23.3-30.4)	27.6 (24.3-31.7)	27.8 (24.4-32)	27.5 (24.2-31.7)
Total cholesterol	5.36 (4.6-6.1)	5.5 (4.8-6.27)	5.5 (4.8-6.3)	5.5 (4.8-6.2)	5.4 (4.7-6.2)
HDL cholesterol	1.5 (1.2-1.8)	1.4 (1.2-1.71)	1.36 (1.1-1.63)	1.3 (1.1-1.6)	1.3 (1.1-1.6)
eGFR	81.4 (67.6-95.4)	82.1 (69.1-95.2)	81.7 (68.8-94.6)	81.9 (68.7-95.1)	82.4 (68.7-95.7)
Diagnoses on or before index date, n (%):					
Atrial fibrillation	555 (1.3%)	3137 (1.0%)	2163 (0.9%)	1027 (1.0%)	840 (0.9%)
Cancer	3895 (8.8%)	19 459 (6.3%)	13 523 (5.9%)	5950 (5.6%)	4694 (5.3%)
Diabetes	1502 (3.4%)	12 125 (3.9%)	11 941 (5.2%)	6022 (5.7%)	5537 (6.2%)
Asthma or atopy	9783 (22.2%)	73 708 (24.0%)	63 179 (27.6%)	33 355 (31.4%)	32 875 (37.1%)
COPD	863 (2.0%)	4417 (1.4%)	4445 (1.9%)	2484 (2.3%)	2812 (3.2%)
Connective tissue disease	1680 (3.8%)	8526 (2.8%)	6236 (2.7%)	2916 (2.7%)	2501 (2.8%)
IBD	704 (1.6%)	3077 (1.0%)	2400 (1.0%)	1249 (1.2%)	1216 (1.4%)
Medication use in the year before index date, n (%):					
Antihypertensives	9988 (22.6%)	73 943 (24.0%)	60 755 (26.6%)	28 752 (27.1%)	23 843 (26.9%)
Statins	1654 (3.7%)	15 864 (5.2%)	15 207 (6.7%)	7503 (7.1%)	6578 (7.4%)

COPD, chronic obstructive pulmonary disease; IBD, inflammatory bowel disease; IQR, interquartile range.

Supplementary Table 5. Characteristics of patients by quintile of lymphocyte count

Category	1 (lowest)	2	3	4	5 (highest)
**Lymphocytes, $\times 10^9$/L**	**<1.45**	**1.45–1.85**	**1.85–2.15**	**2.15–2.55**	**≥2.55**
N patients	133 220	192 867	147 913	144 580	156 651
Women, n (%)	80 119 (60.1%)	116 445 (60.4%)	88 563 (59.9%)	85 741 (59.3%)	91 088 (58.1%)
Age, median (IQR)	55.8 (43.4–70.4)	52.2 (41.3–64.7)	51.6 (40.9–63.4)	51.4 (40.7–62.9)	51.9 (41.1–62.9)
Most deprived quintile, n (%)	18 982 (14.3%)	28 914 (15.0%)	24 222 (16.4%)	26 314 (18.3%)	34 539 (22.1%)
Ethnicity, n (%):					
White	80 558 (95.3%)	110 998 (94.0%)	83 915 (92.9%)	81 626 (91.8%)	87 937 (89.8%)
South Asian	1134 (1.3%)	2341 (2.0%)	2349 (2.6%)	2935 (3.3%)	4691 (4.8%)
Black	1214 (1.4%)	2205 (1.9%)	1927 (2.1%)	2041 (2.3%)	2587 (2.6%)
Other	1598 (1.9%)	2571 (2.2%)	2142 (2.4%)	2301 (2.6%)	2708 (2.8%)
Missing	48 716 (36.6%)	74 752 (38.8%)	57 580 (38.9%)	55 677 (38.5%)	58 728 (37.5%)
Complete blood count parameters on index date, median (IQR):					
Neutrophils, $\times 10^9$/L	3.6 (2.76–4.85)	3.6 (2.86–4.69)	3.79 (3–4.8)	4 (3.18–5.01)	4.4 (3.5–5.6)
Eosinophils, $\times 10^9$/L	0.1 (0.1–0.2)	0.14 (0.1–0.2)	0.18 (0.1–0.24)	0.2 (0.1–0.3)	0.2 (0.11–0.3)
Monocytes, $\times 10^9$/L	0.4 (0.3–0.53)	0.43 (0.35–0.57)	0.5 (0.4–0.6)	0.5 (0.4–0.6)	0.58 (0.44–0.7)
Basophils, $\times 10^9$/L	0.01 (0–0.04)	0.02 (0–0.06)	0.02 (0–0.07)	0.03 (0–0.09)	0.04 (0–0.1)
Haemoglobin, g/dL	13.6 (12.5–14.6)	13.8 (12.9–14.8)	13.9 (13–14.9)	14 (13.1–15)	14.2 (13.3–15.2)
Platelets, $\times 10^9$/L	242 (204–289)	252 (215–295)	260 (223–303)	267 (229–310)	278 (238–326)
Smoking status, n (%):					
Never	76 478 (61.6%)	106 318 (58.3%)	75 959 (54.0%)	68 025 (49.3%)	59 922 (40.0%)
Ex	30 682 (24.7%)	45 532 (25.0%)	34 970 (24.8%)	32 899 (23.9%)	33 116 (22.1%)
Current	16 925 (13.6%)	30 601 (16.8%)	29 857 (21.2%)	36 957 (26.8%)	56 681 (37.9%)
Missing	9135 (6.9%)	10416 (5.4%)	7127 (4.8%)	6699 (4.6%)	6932 (4.4%)
Most recent value within one year prior to index date, median (IQR):					
Systolic blood pressure	136 (120–150)	135 (120–150)	136 (121–150)	136 (122–150)	138 (124–150)
Body mass index	25.5 (22.5–29)	26.4 (23.4–30.1)	27.1 (23.9–31.1)	27.6 (24.3–31.7)	28.4 (24.8–32.8)
Total cholesterol	5.3 (4.6–6.1)	5.4 (4.7–6.2)	5.5 (4.8–6.2)	5.5 (4.8–6.3)	5.6 (4.8–6.4)
HDL cholesterol	1.5 (1.2–1.8)	1.4 (1.2–1.71)	1.4 (1.15–1.7)	1.35 (1.1–1.61)	1.3 (1.1–1.55)
eGFR	78.9 (65.1–92.1)	81.4 (68.6–94.3)	82.5 (69.6–95.4)	83.1 (70.1–96.1)	83.6 (70.4–96.8)
Diagnoses on or before index date, n (%):					
Atrial fibrillation	2358 (1.8%)	1891 (1.0%)	1284 (0.9%)	1068 (0.7%)	1121 (0.7%)
Cancer	13109 (9.8%)	11771 (6.1%)	7860 (5.3%)	6997 (4.8%)	7784 (5.0%)
Diabetes	5807 (4.4%)	7915 (4.1%)	6478 (4.4%)	7020 (4.9%)	9907 (6.3%)
Asthma	15 801 (11.9%)	23 270 (12.1%)	18 444 (12.5%)	18 345 (12.7%)	20 937 (13.4%)
COPD	3250 (2.4%)	3246 (1.7%)	2390 (1.6%)	2474 (1.7%)	3661 (2.3%)
Connective tissue disease	5629 (4.2%)	5370 (2.8%)	3645 (2.5%)	3354 (2.3%)	3861 (2.5%)
IBD	2337 (1.8%)	2144 (1.1%)	1453 (1.0%)	1326 (0.9%)	1386 (0.9%)
Medication use in the year before index date, n (%):					
Antihypertensives	35 031 (26.3%)	46 699 (24.2%)	36 257 (24.5%)	36 393 (25.2%)	42 901 (27.4%)
Statins	6638 (5.0%)	10 343 (5.4%)	8638 (5.8%)	9163 (6.3%)	12 024 (7.7%)

COPD, chronic obstructive pulmonary disease; IBD, inflammatory bowel disease; IQR, interquartile range.

Supplementary Table 6. Prevalence of acute conditions on date of blood testing by category of eosinophil count

**Category**<br>**Eosinophil count, $\times 10^9$/L**	**1 (lowest)**<br>< 0.05	**2**<br>**0.05–0.15**	**3**<br>**0.15–0.25**	**4**<br>**0.25–0.35**	**5 (highest)**<br>≥ 0.35
In hospital on date of blood test	662 (1.5%)	1177 (0.4%)	670 (0.3%)	291 (0.3%)	280 (0.3%)
Vaccination within previous 7 days	399 (0.9%)	2998 (1.0%)	2446 (1.1%)	1276 (1.2%)	1052 (1.2%)
Anaemia diagnosis within 30 days before	393 (0.9%)	1617 (0.5%)	940 (0.4%)	407 (0.4%)	400 (0.5%)
Infection diagnosis within 30 days before	3859 (8.7%)	20 129 (6.5%)	15 622 (6.8%)	7543 (7.1%)	7098 (8.0%)
Infective symptoms within 30 days before	3439 (7.8%)	16 166 (5.3%)	11 902 (5.2%)	5719 (5.4%)	5952 (6.7%)
Prior diagnosis of myelodysplastic syndrome	79 (0.2%)	117 (0.0%)	55 (0.0%)	31 (0.0%)	24 (0.0%)
Prior diagnosis of haemoglobinopathy	200 (0.5%)	1082 (0.4%)	688 (0.3%)	352 (0.3%)	327 (0.4%)
Chemotherapy or G-CSF within 6 months prior	561 (1.3%)	780 (0.3%)	390 (0.2%)	153 (0.1%)	172 (0.2%)
Methotrexate within 3 months prior	203 (0.5%)	1175 (0.4%)	966 (0.4%)	391 (0.4%)	342 (0.4%)
Steroid within 3 months prior	2304 (5.2%)	7244 (2.4%)	5473 (2.4%)	2849 (2.7%)	3306 (3.7%)
Other immune drug within 3 months prior	993 (2.3%)	3308 (1.1%)	2182 (1.0%)	992 (0.9%)	918 (1.0%)
Prior diagnosis of cancer	3895 (8.8%)	19 459 (6.3%)	13 523 (5.9%)	5950 (5.6%)	4694 (5.3%)
Any acute condition	12 068 (27.4%)	58 821 (19.1%)	43 324 (18.9%)	20 619 (19.4%)	19 347 (21.8%)

Supplementary Table 7. Prevalence of acute conditions on date of blood testing by quintile of lymphocyte count

**Quintile**<br>**Lymphocyte count, $\times 10^9$/L**	**1 (lowest)**<br>**<1.45**	**2**<br>**1.45–1.85**	**3**<br>**1.85–2.15**	**4**<br>**2.15–2.55**	**5 (highest)**<br>**≥2.55**
In hospital on date of blood test	1173 (0.9%)	712 (0.4%)	432 (0.3%)	339 (0.2%)	424 (0.3%)
Vaccination within previous 7 days	1619 (1.2%)	2064 (1.1%)	1426 (1.0%)	1450 (1.0%)	1612 (1.0%)
Anaemia diagnosis within 30 days before	1254 (0.9%)	1019 (0.5%)	615 (0.4%)	468 (0.3%)	401 (0.3%)
Infection diagnosis within 30 days before	9865 (7.4%)	12 649 (6.6%)	9970 (6.7%)	9853 (6.8%)	11 914 (7.6%)
Infective symptoms within 30 days before	8351 (6.3%)	10 300 (5.3%)	7660 (5.2%)	7710 (5.3%)	9157 (5.8%)
Prior diagnosis of myelodysplastic syndrome	143 (0.1%)	61 (0.0%)	43 (0.0%)	21 (0.0%)	38 (0.0%)
Prior diagnosis of haemoglobinopathy	392 (0.3%)	592 (0.3%)	498 (0.3%)	507 (0.4%)	660 (0.4%)
Chemotherapy or G-CSF within 6 months prior	1127 (0.8%)	386 (0.2%)	203 (0.1%)	162 (0.1%)	178 (0.1%)
Methotrexate within 3 months prior	988 (0.7%)	777 (0.4%)	493 (0.3%)	414 (0.3%)	405 (0.3%)
Steroid within 3 months prior	5096 (3.8%)	4379 (2.3%)	3210 (2.2%)	3309 (2.3%)	5182 (3.3%)
Other immune drug within 3 months prior	3026 (2.3%)	2090 (1.1%)	1214 (0.8%)	1055 (0.7%)	1008 (0.6%)
Prior diagnosis of cancer	13 109 (9.8%)	11 771 (6.1%)	7860 (5.3%)	6997 (4.8%)	7784 (5.0%)
Any acute condition	34 023 (25.5%)	36 887 (19.1%)	26 695 (18.0%)	25 854 (17.9%)	30 720 (19.6%)

Supplementary Table 8. Endpoints by category of eosinophil count

Category<br>Eosinophil count, $\times 10^9$/L	1 (lowest)<br>< 0.05	2<br>0.05–0.15	3<br>0.15–0.25	4<br>0.25–0.35	5 (highest)<br>≥ 0.35
Initial presentation of cardiovascular disease					
Stable angina	440 (13.6%)	3494 (17.3%)	2975 (18.0%)	1475 (18.2%)	1164 (16.8%)
Unstable angina	151 (4.7%)	1061 (5.3%)	876 (5.3%)	441 (5.4%)	351 (5.1%)
Coronary disease not further specified	193 (6.0%)	1805 (8.9%)	1546 (9.3%)	772 (9.5%)	607 (8.8%)
Non-fatal myocardial infarction	335 (10.4%)	2219 (11.0%)	2039 (12.3%)	1062 (13.1%)	911 (13.2%)
Unheralded coronary death	240 (7.4%)	1119 (5.5%)	895 (5.4%)	436 (5.4%)	400 (5.8%)
Heart failure	545 (16.9%)	2391 (11.8%)	1761 (10.6%)	794 (9.8%)	733 (10.6%)
Ventricular arrhythmia or sudden cardiac death	60 (1.9%)	288 (1.4%)	215 (1.3%)	91 (1.1%)	93 (1.3%)
Transient ischaemic attack	311 (9.6%)	1931 (9.6%)	1538 (9.3%)	704 (8.7%)	592 (8.6%)
Ischaemic stroke	223 (6.9%)	1418 (7.0%)	1082 (6.5%)	542 (6.7%)	425 (6.1%)
Stroke not further specified	307 (9.5%)	1638 (8.1%)	1181 (7.1%)	558 (6.9%)	507 (7.3%)
Subarachnoid haemorrhage	38 (1.2%)	246 (1.2%)	155 (0.9%)	74 (0.9%)	64 (0.9%)
Intracerebral haemorrhage	91 (2.8%)	432 (2.1%)	303 (1.8%)	114 (1.4%)	120 (1.7%)
Peripheral arterial disease	240 (7.4%)	1708 (8.5%)	1576 (9.5%)	799 (9.9%)	743 (10.7%)
Abdominal aortic aneurysm	58 (1.8%)	448 (2.2%)	420 (2.5%)	231 (2.9%)	209 (3.0%)
Total	3232	20 198	16 562	8093	6919
Other deaths					
Cancers	2132 (54.9%)	6858 (53.7%)	4578 (55.3%)	2105 (53.9%)	1920 (51.1%)
Dementia	167 (4.3%)	682 (5.3%)	370 (4.5%)	204 (5.2%)	182 (4.8%)
Pneumonia	277 (7.1%)	790 (6.2%)	498 (6.0%)	240 (6.2%)	259 (6.9%)
Chronic obstructive pulmonary disease	129 (3.3%)	479 (3.8%)	352 (4.3%)	171 (4.4%)	195 (5.2%)
Liver disease	127 (3.3%)	367 (2.9%)	212 (2.6%)	86 (2.2%)	77 (2.1%)
Other causes of death	1052 (27.1%)	3597 (28.2%)	2267 (27.4%)	1096 (28.1%)	1122 (29.9%)
Total	3884	12 773	8277	3902	3755

Supplementary Table 9. Endpoints by quintile of lymphocyte count

**Quintile**	**1 (lowest)**	**2**	**3**	**4**	**5 (highest)**
**Lymphocyte count, $\times 10^9$/L**	**<1.45**	**1.45–1.85**	**1.85–2.15**	**2.15–2.55**	**$\geq$2.55**
Initial presentation of cardiovascular disease					
Stable angina	1596 (13.4%)	2262 (17.5%)	1710 (18.3%)	1789 (19.1%)	2191 (19.0%)
Unstable angina	451 (3.8%)	662 (5.1%)	502 (5.4%)	581 (6.2%)	684 (5.9%)
Coronary disease not further specified	794 (6.7%)	1150 (8.9%)	929 (9.9%)	930 (9.9%)	1120 (9.7%)
Non-fatal myocardial infarction	1165 (9.8%)	1512 (11.7%)	1146 (12.2%)	1173 (12.5%)	1570 (13.6%)
Unheralded coronary death	836 (7.0%)	737 (5.7%)	512 (5.5%)	468 (5.0%)	537 (4.7%)
Heart failure	2009 (16.9%)	1523 (11.8%)	892 (9.5%)	864 (9.2%)	936 (8.1%)
Ventricular arrhythmia or sudden cardiac death	184 (1.5%)	164 (1.3%)	141 (1.5%)	102 (1.1%)	156 (1.4%)
Transient ischaemic attack	1154 (9.7%)	1233 (9.6%)	871 (9.3%)	855 (9.1%)	963 (8.4%)
Ischaemic stroke	873 (7.3%)	914 (7.1%)	644 (6.9%)	576 (6.2%)	683 (5.9%)
Stroke not further specified	1202 (10.1%)	982 (7.6%)	647 (6.9%)	648 (6.9%)	712 (6.2%)
Subarachnoid haemorrhage	117 (1.0%)	127 (1.0%)	104 (1.1%)	89 (1.0%)	140 (1.2%)
Intracerebral haemorrhage	307 (2.6%)	261 (2.0%)	163 (1.7%)	160 (1.7%)	169 (1.5%)
Peripheral arterial disease	920 (7.7%)	1053 (8.2%)	827 (8.8%)	897 (9.6%)	1369 (11.9%)
Abdominal aortic aneurysm	274 (2.3%)	310 (2.4%)	274 (2.9%)	228 (2.4%)	280 (2.4%)
Total	11 882	12 890	9362	9360	11 510
Other deaths					
Cancers	5898 (51.4%)	3935 (52.0%)	2600 (55.2%)	2328 (56.4%)	2832 (60.0%)
Dementia	654 (5.7%)	426 (5.6%)	202 (4.3%)	173 (4.2%)	150 (3.2%)
Pneumonia	873 (7.6%)	501 (6.6%)	258 (5.5%)	206 (5.0%)	226 (4.8%)
Chronic obstructive pulmonary disease	471 (4.1%)	280 (3.7%)	186 (3.9%)	165 (4.0%)	224 (4.7%)
Liver disease	289 (2.5%)	187 (2.5%)	143 (3.0%)	122 (3.0%)	128 (2.7%)
Other causes of death	3283 (28.6%)	2232 (29.5%)	1325 (28.1%)	1134 (27.5%)	1160 (24.6%)
Total	11 468	7561	4714	4128	4720

Supplementary Figures

Supplementary Figure 1. Histogram showing distribution of eosinophil counts and category cutpoints

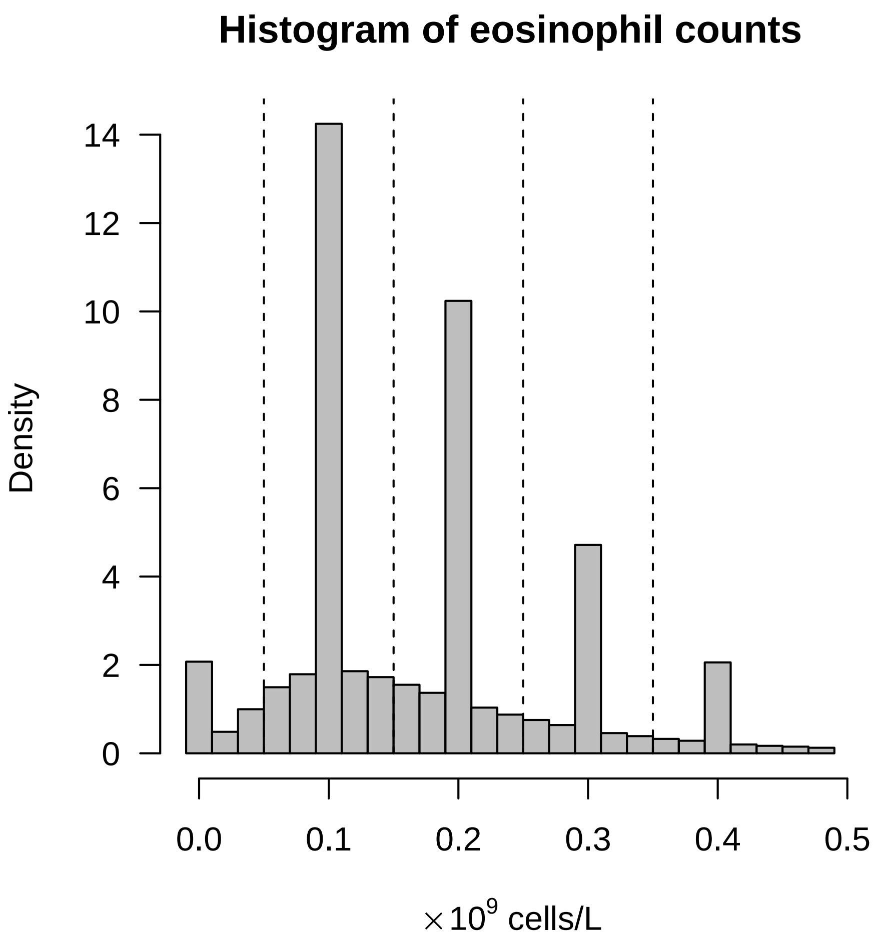

Supplementary Figure 2. Patient flow diagram

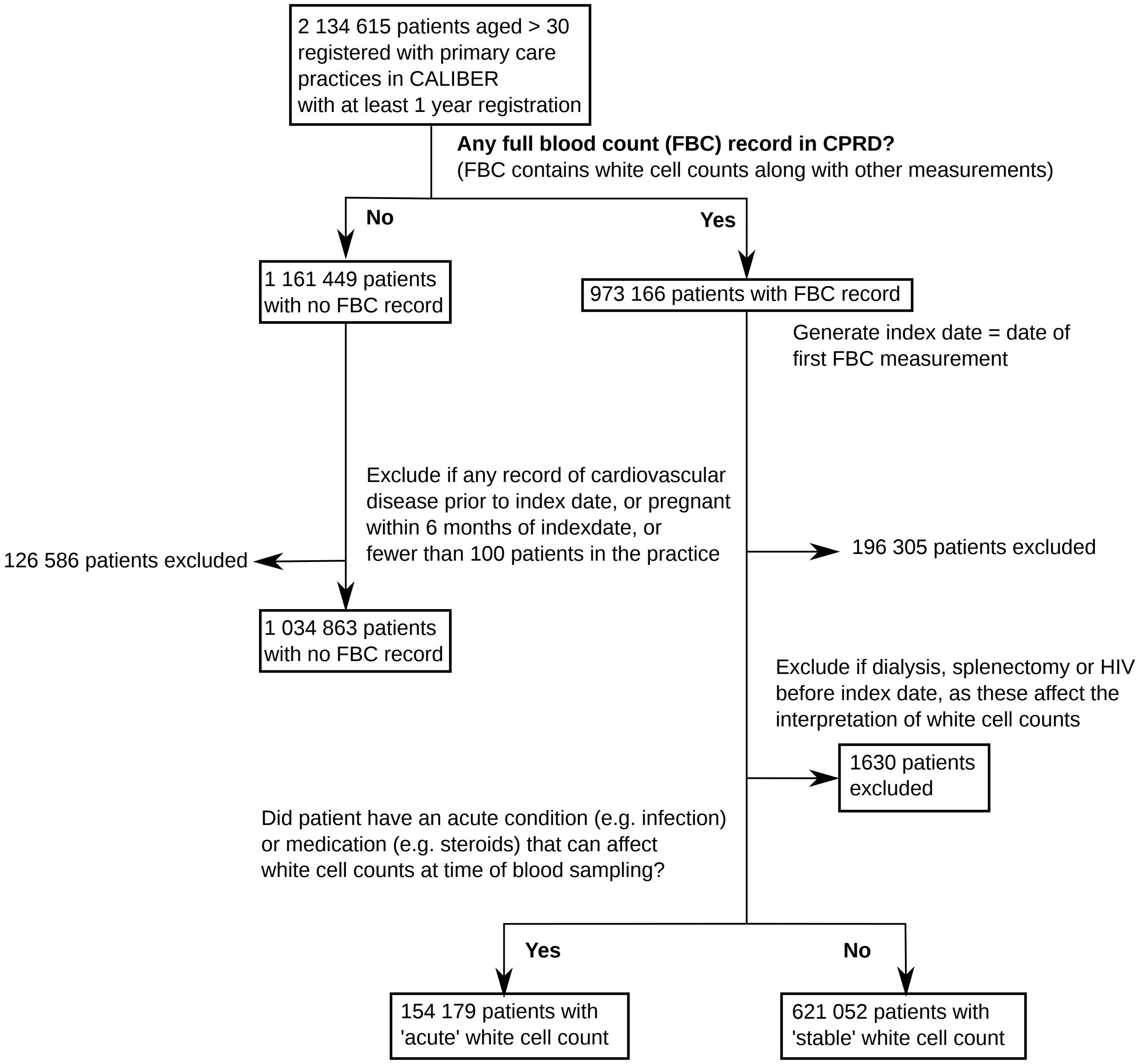

Supplementary Figure 3. Multivariable adjusted association of eosinophil categories with different initial presentations of cardiovascular disease

Hazard ratios are adjusted for age, sex, deprivation, ethnicity, smoking, diabetes, systolic blood pressure, body mass index, total cholesterol, HDL cholesterol, eGFR, atrial fibrillation, autoimmune conditions, inflammatory bowel disease, COPD, cancer, statin use, blood pressure medication and acute conditions at the time of blood testing. P values * < 0.05, ** < 0.01, *** < 0.001

Initial presentation	*Hazard ratio (95% CI)*
Stable angina	
<0.05	0.93 (0.84, 1.03)
0.05−0.15	0.96 (0.91, 1.01)
0.15−0.25	1 (reference)
0.25−0.35	1.05 (0.98, 1.12)
≥ 0.35	1.00 (0.94, 1.07)
Unstable angina	
<0.05	1.07 (0.90, 1.28)
0.05−0.15	1.00 (0.91, 1.09)
0.15−0.25	1 (reference)
0.25−0.35	1.05 (0.94, 1.18)
≥ 0.35	1.00 (0.88, 1.13)
Coronary disease not further specified	
<0.05	0.85 (0.73, 0.99) *
0.05−0.15	0.99 (0.92, 1.06)
0.15−0.25	1 (reference)
0.25−0.35	1.04 (0.95, 1.13)
≥ 0.35	0.98 (0.90, 1.08)
Non−fatal myocardial infarction	
<0.05	1.00 (0.89, 1.12)
0.05−0.15	0.93 (0.87, 0.98) *
0.15−0.25	1 (reference)
0.25−0.35	1.05 (0.97, 1.13)
≥ 0.35	1.04 (0.96, 1.13)
Unheralded coronary death	
<0.05	1.29 (1.12, 1.50) ***
0.05−0.15	1.00 (0.91, 1.09)
0.15−0.25	1 (reference)
0.25−0.35	0.99 (0.88, 1.11)
≥ 0.35	1.03 (0.91, 1.16)
Heart failure	
<0.05	1.39 (1.26, 1.54) ***
0.05−0.15	1.06 (1.00, 1.13)
0.15−0.25	1 (reference)
0.25−0.35	0.94 (0.87, 1.03)
≥ 0.35	1.01 (0.93, 1.11)
Ventricular arrhythmia or sudden cardiac death	
<0.05	1.49 (1.12, 2.00) **
0.05−0.15	1.06 (0.88, 1.26)
0.15−0.25	1 (reference)
0.25−0.35	0.86 (0.67, 1.10)
≥ 0.35	1.05 (0.82, 1.34)

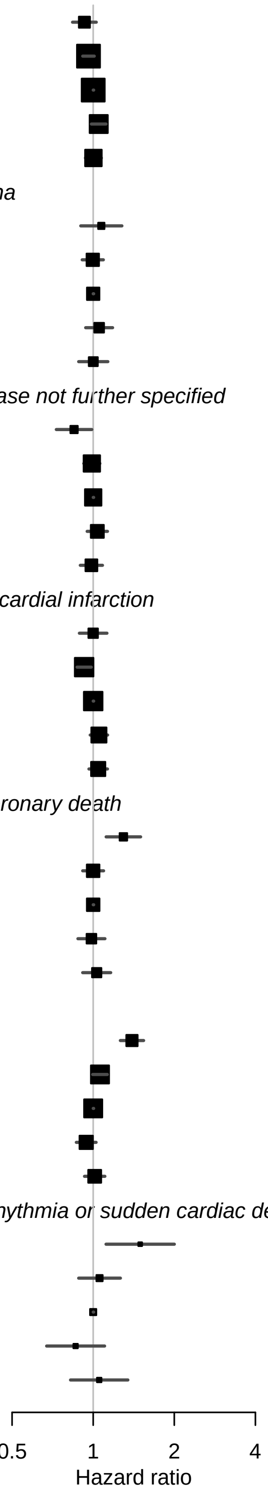

Initial presentation	*Hazard ratio (95% CI)*
Transient ischaemic attack	
<0.05	0.99 (0.88, 1.13)
0.05−0.15	0.95 (0.89, 1.02)
0.15−0.25	1 (reference)
0.25−0.35	0.99 (0.90, 1.08)
≥ 0.35	1.00 (0.91, 1.10)
Ischaemic stroke	
<0.05	1.02 (0.88, 1.19)
0.05−0.15	1.00 (0.93, 1.09)
0.15−0.25	1 (reference)
0.25−0.35	1.06 (0.96, 1.18)
≥ 0.35	0.99 (0.88, 1.11)
Stroke not further specified	
<0.05	1.11 (0.97, 1.26)
0.05−0.15	1.01 (0.94, 1.09)
0.15−0.25	1 (reference)
0.25−0.35	1.02 (0.92, 1.12)
≥ 0.35	1.09 (0.98, 1.21)
Subarachnoid haemorrhage	
<0.05	1.30 (0.90, 1.86)
0.05−0.15	1.20 (0.98, 1.47)
0.15−0.25	1 (reference)
0.25−0.35	1.03 (0.78, 1.37)
≥ 0.35	1.10 (0.82, 1.48)
Intracerebral haemorrhage	
<0.05	1.33 (1.05, 1.69) *
0.05−0.15	1.04 (0.90, 1.21)
0.15−0.25	1 (reference)
0.25−0.35	0.81 (0.66, 1.01)
≥ 0.35	1.02 (0.83, 1.26)
Peripheral arterial disease	
<0.05	0.87 (0.76, 1.00) *
0.05−0.15	0.92 (0.85, 0.98) *
0.15−0.25	1 (reference)
0.25−0.35	1.02 (0.94, 1.12)
≥ 0.35	1.10 (1.00, 1.20) *
Abdominal aortic aneurysm	
<0.05	0.80 (0.60, 1.05)
0.05−0.15	0.94 (0.83, 1.08)
0.15−0.25	1 (reference)
0.25−0.35	1.06 (0.90, 1.24)
≥ 0.35	1.05 (0.89, 1.25)

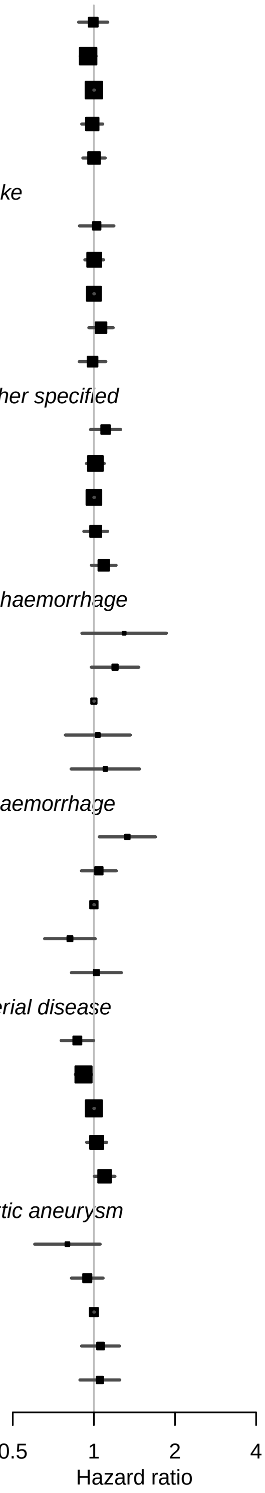

Supplementary Figure 4. Multivariable adjusted association of lymphocyte quintiles with different initial presentations of cardiovascular disease

Hazard ratios are adjusted for age, sex, deprivation, ethnicity, smoking, diabetes, systolic blood pressure, blood pressure medication, body mass index, total cholesterol, HDL cholesterol, statin use, eGFR, atrial fibrillation, autoimmune conditions, inflammatory bowel disease, COPD, cancer, and acute conditions at the time of blood testing. P values * < 0.05, ** < 0.01, *** < 0.001

Initial presentation	*Hazard ratio (95% CI)*
Stable angina	
<1.45	1.01 (0.95, 1.09)
1.45−1.85	1.03 (0.96, 1.09)
1.85−2.15	1 (reference)
2.15−2.55	1.04 (0.98, 1.12)
≥ 2.55	1.08 (1.01, 1.15) *
Unstable angina	
<1.45	0.99 (0.87, 1.13)
1.45−1.85	1.03 (0.92, 1.16)
1.85−2.15	1 (reference)
2.15−2.55	1.16 (1.03, 1.30) *
≥ 2.55	1.15 (1.02, 1.30) *
Coronary disease not further specified	
<1.45	0.92 (0.83, 1.01)
1.45−1.85	0.96 (0.88, 1.04)
1.85−2.15	1 (reference)
2.15−2.55	1.01 (0.92, 1.10)
≥ 2.55	1.02 (0.93, 1.11)
Non−fatal myocardial infarction	
<1.45	1.02 (0.94, 1.11)
1.45−1.85	1.02 (0.94, 1.10)
1.85−2.15	1 (reference)
2.15−2.55	1.02 (0.94, 1.10)
≥ 2.55	1.12 (1.03, 1.21) **
Unheralded coronary death	
<1.45	1.27 (1.13, 1.42) ***
1.45−1.85	1.03 (0.92, 1.15)
1.85−2.15	1 (reference)
2.15−2.55	0.95 (0.83, 1.07)
≥ 2.55	0.92 (0.81, 1.04)
Heart failure	
<1.45	1.61 (1.48, 1.74) ***
1.45−1.85	1.19 (1.09, 1.29) ***
1.85−2.15	1 (reference)
2.15−2.55	1.02 (0.93, 1.12)
≥ 2.55	0.97 (0.88, 1.06)
Ventricular arrhythmia or sudden cardiac death	
<1.45	1.22 (0.97, 1.52)
1.45−1.85	0.85 (0.68, 1.07)
1.85−2.15	1 (reference)
2.15−2.55	0.75 (0.58, 0.96) *
≥ 2.55	1.01 (0.80, 1.28)

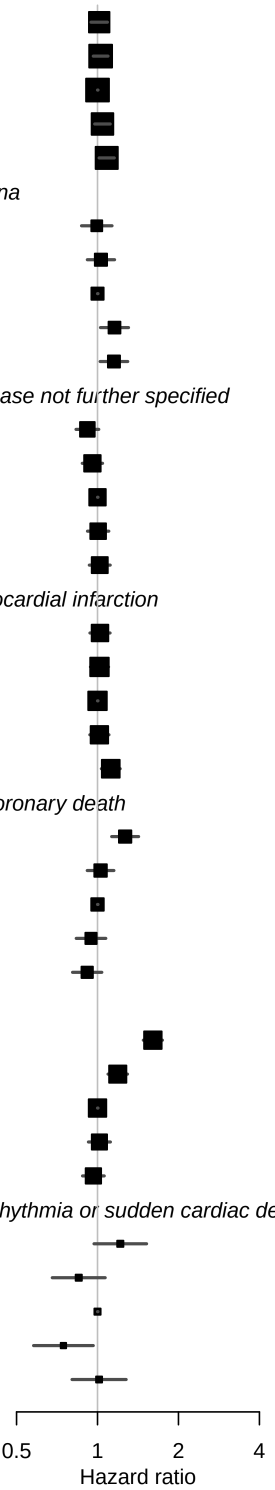

Initial presentation	*Hazard ratio (95% CI)*
Transient ischaemic attack	
<1.45	1.11 (1.02, 1.22) *
1.45−1.85	1.01 (0.93, 1.10)
1.85−2.15	1 (reference)
2.15−2.55	1.03 (0.94, 1.13)
≥ 2.55	1.05 (0.96, 1.15)
Ischaemic stroke	
<1.45	1.08 (0.97, 1.20)
1.45−1.85	1.00 (0.91, 1.11)
1.85−2.15	1 (reference)
2.15−2.55	0.94 (0.84, 1.05)
≥ 2.55	1.00 (0.89, 1.11)
Stroke not further specified	
<1.45	1.33 (1.20, 1.47) ***
1.45−1.85	1.04 (0.94, 1.14)
1.85−2.15	1 (reference)
2.15−2.55	1.06 (0.95, 1.18)
≥ 2.55	1.06 (0.95, 1.18)
Subarachnoid haemorrhage	
<1.45	1.30 (0.99, 1.70)
1.45−1.85	0.97 (0.74, 1.25)
1.85−2.15	1 (reference)
2.15−2.55	0.82 (0.62, 1.09)
≥ 2.55	1.09 (0.84, 1.41)
Intracerebral haemorrhage	
<1.45	1.46 (1.20, 1.77) ***
1.45−1.85	1.11 (0.91, 1.36)
1.85−2.15	1 (reference)
2.15−2.55	1.04 (0.84, 1.30)
≥ 2.55	1.02 (0.82, 1.27)
Peripheral arterial disease	
<1.45	1.10 (1.00, 1.22) *
1.45−1.85	0.99 (0.90, 1.09)
1.85−2.15	1 (reference)
2.15−2.55	1.06 (0.97, 1.17)
≥ 2.55	1.26 (1.15, 1.38) ***
Abdominal aortic aneurysm	
<1.45	0.82 (0.69, 0.97) *
1.45−1.85	0.83 (0.70, 0.98) *
1.85−2.15	1 (reference)
2.15−2.55	0.88 (0.74, 1.05)
≥ 2.55	0.89 (0.75, 1.05)

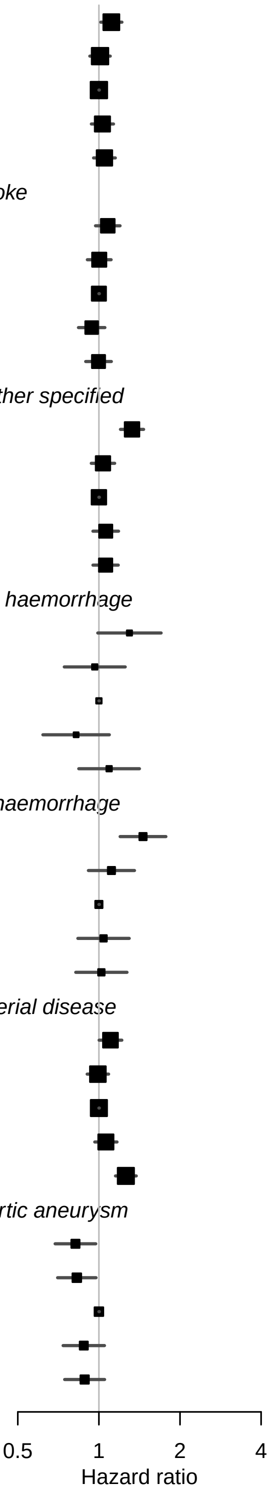

Supplementary Figure 5. Association of eosinophil categories with different initial presentations of cardiovascular disease, adjusted for lymphocyte count

Hazard ratios are adjusted for lymphocyte count quintile, age, sex, deprivation, ethnicity, smoking, diabetes, systolic blood pressure, body mass index, total cholesterol, HDL cholesterol, eGFR, atrial fibrillation, autoimmune conditions, inflammatory bowel disease, COPD, cancer, statin use, blood pressure medication and acute conditions at the time of blood testing. P values * < 0.05, ** < 0.01, *** < 0.001

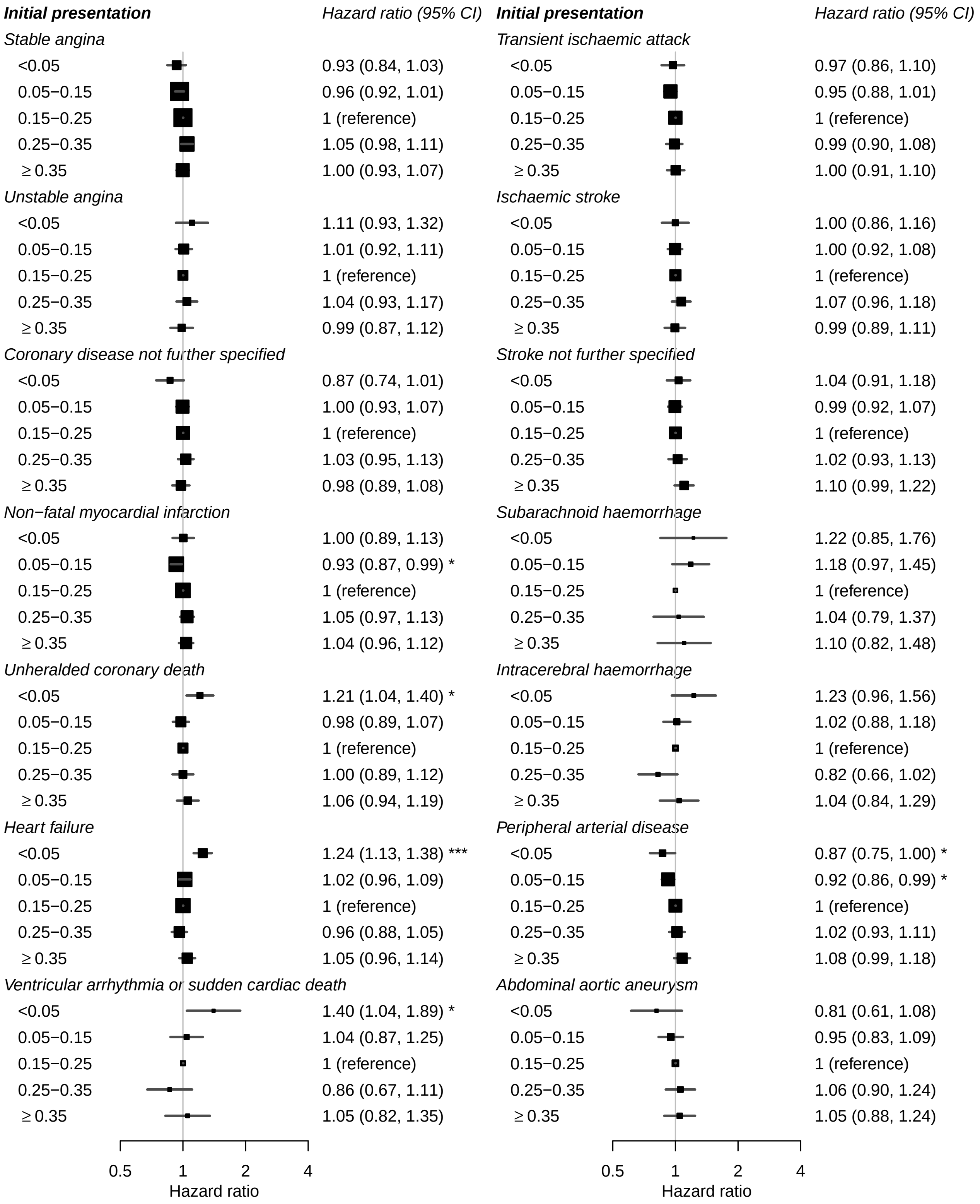

Supplementary Figure 6. Association of lymphocyte quintiles with different initial presentations of cardiovascular disease, adjusted for eosinophil count

Hazard ratios are adjusted for eosinophil count category, age, sex, deprivation, ethnicity, smoking, diabetes, systolic blood pressure, blood pressure medication, body mass index, total cholesterol, HDL cholesterol, statin use, eGFR, atrial fibrillation, autoimmune conditions, inflammatory bowel disease, COPD, cancer, and acute conditions at the time of blood testing. P values * < 0.05, ** < 0.01, *** < 0.001

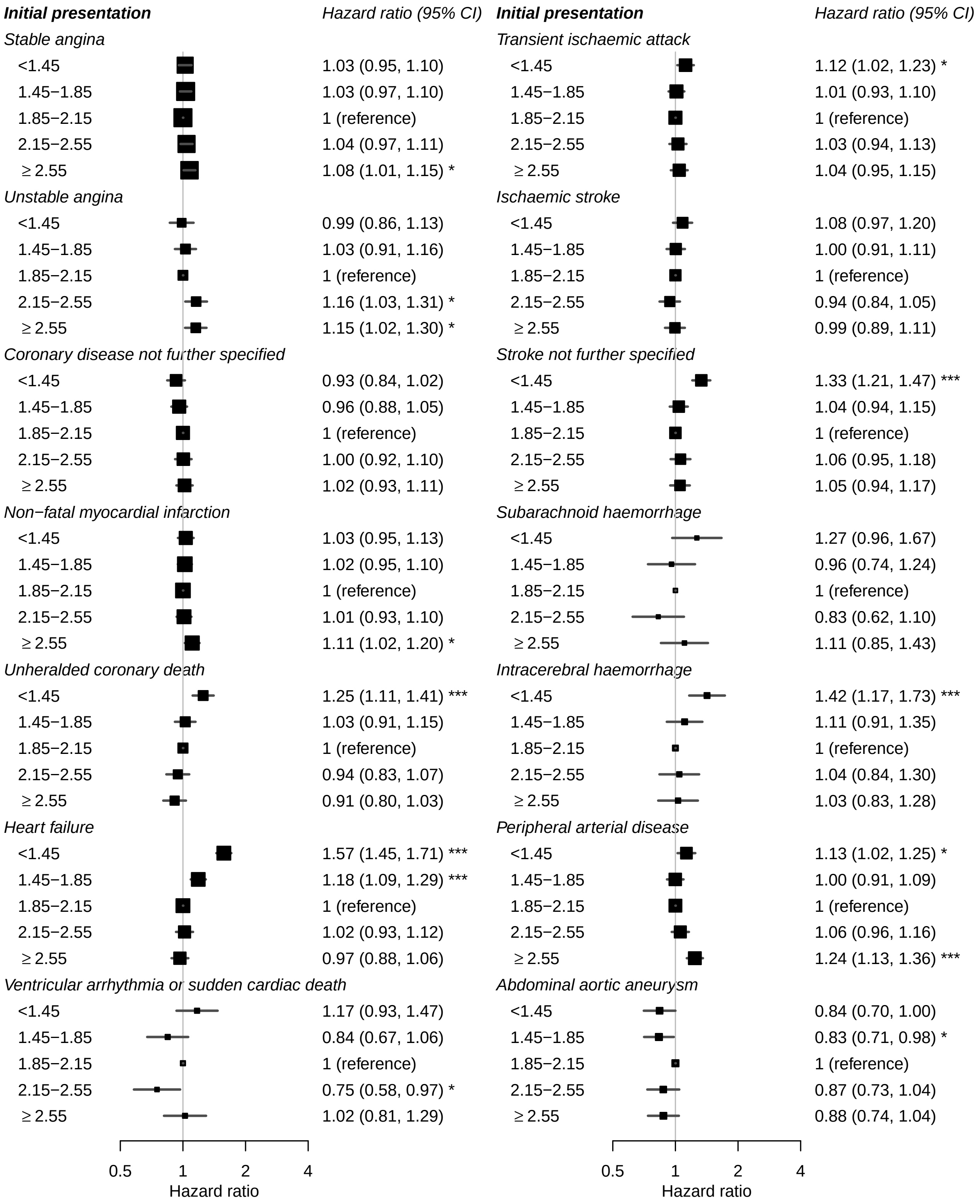

Supplementary Figure 7. Age and sex adjusted association of eosinophil categories with different initial presentations of cardiovascular diseases

P values * < 0.05, ** < 0.01, *** < 0.001

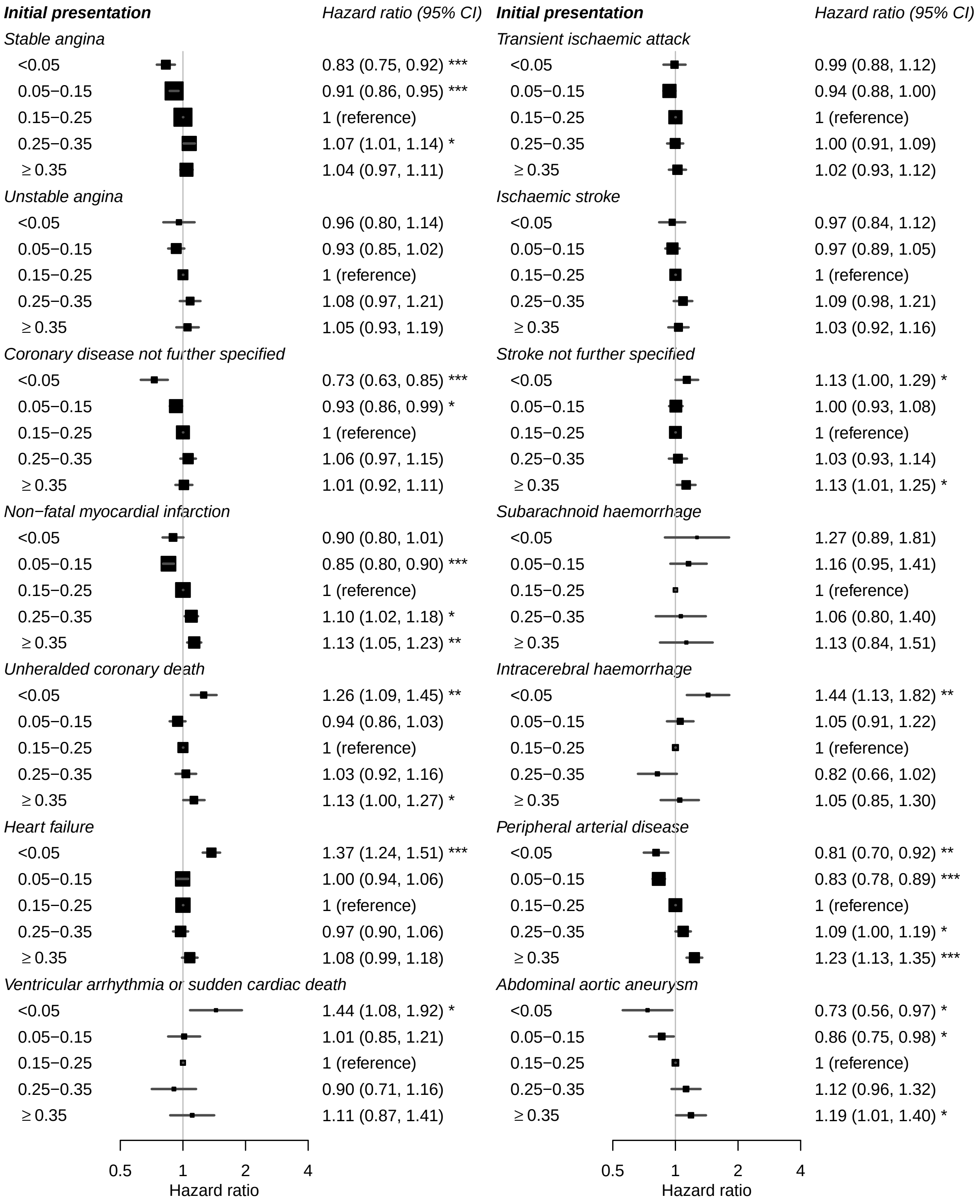

Supplementary Figure 8. Multivariable adjusted association of eosinophil categories with different initial presentations of cardiovascular disease, by time after measurement

Hazard ratios are adjusted for age, sex, deprivation, ethnicity, smoking, diabetes, systolic blood pressure, body mass index, total cholesterol, HDL cholesterol, eGFR, atrial fibrillation, autoimmune conditions, inflammatory bowel disease, COPD, cancer, statin use, blood pressure medication and acute conditions at the time of blood testing. P values * < 0.05, ** < 0.01, *** < 0.001

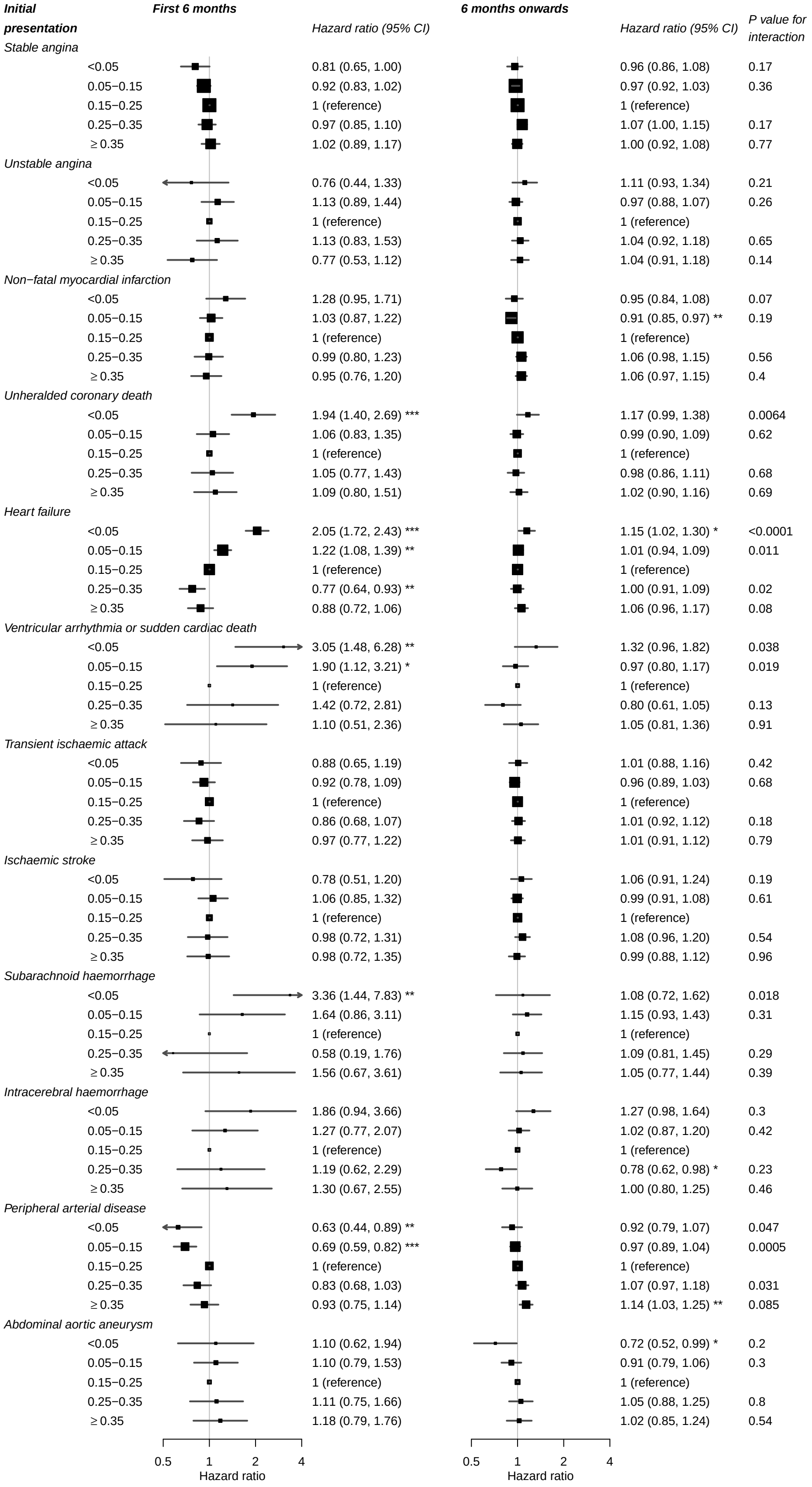

Initial presentation	*First 6 months* Hazard ratio (95% CI)	*6 months onwards* Hazard ratio (95% CI)	*P value for interaction*
Stable angina			
<0.05	0.81 (0.65, 1.00)	0.96 (0.86, 1.08)	0.17
0.05−0.15	0.92 (0.83, 1.02)	0.97 (0.92, 1.03)	0.36
0.15−0.25	1 (reference)	1 (reference)	
0.25−0.35	0.97 (0.85, 1.10)	1.07 (1.00, 1.15)	0.17
≥ 0.35	1.02 (0.89, 1.17)	1.00 (0.92, 1.08)	0.77
Unstable angina			
<0.05	0.76 (0.44, 1.33)	1.11 (0.93, 1.34)	0.21
0.05−0.15	1.13 (0.89, 1.44)	0.97 (0.88, 1.07)	0.26
0.15−0.25	1 (reference)	1 (reference)	
0.25−0.35	1.13 (0.83, 1.53)	1.04 (0.92, 1.18)	0.65
≥ 0.35	0.77 (0.53, 1.12)	1.04 (0.91, 1.18)	0.14
Non−fatal myocardial infarction			
<0.05	1.28 (0.95, 1.71)	0.95 (0.84, 1.08)	0.07
0.05−0.15	1.03 (0.87, 1.22)	0.91 (0.85, 0.97) **	0.19
0.15−0.25	1 (reference)	1 (reference)	
0.25−0.35	0.99 (0.80, 1.23)	1.06 (0.98, 1.15)	0.56
≥ 0.35	0.95 (0.76, 1.20)	1.06 (0.97, 1.15)	0.4
Unheralded coronary death			
<0.05	1.94 (1.40, 2.69) ***	1.17 (0.99, 1.38)	0.0064
0.05−0.15	1.06 (0.83, 1.35)	0.99 (0.90, 1.09)	0.62
0.15−0.25	1 (reference)	1 (reference)	
0.25−0.35	1.05 (0.77, 1.43)	0.98 (0.86, 1.11)	0.68
≥ 0.35	1.09 (0.80, 1.51)	1.02 (0.90, 1.16)	0.69
Heart failure			
<0.05	2.05 (1.72, 2.43) ***	1.15 (1.02, 1.30) *	<0.0001
0.05−0.15	1.22 (1.08, 1.39) **	1.01 (0.94, 1.09)	0.011
0.15−0.25	1 (reference)	1 (reference)	
0.25−0.35	0.77 (0.64, 0.93) **	1.00 (0.91, 1.09)	0.02
≥ 0.35	0.88 (0.72, 1.06)	1.06 (0.96, 1.17)	0.08
Ventricular arrhythmia or sudden cardiac death			
<0.05	3.05 (1.48, 6.28) **	1.32 (0.96, 1.82)	0.038
0.05−0.15	1.90 (1.12, 3.21) *	0.97 (0.80, 1.17)	0.019
0.15−0.25	1 (reference)	1 (reference)	
0.25−0.35	1.42 (0.72, 2.81)	0.80 (0.61, 1.05)	0.13
≥ 0.35	1.10 (0.51, 2.36)	1.05 (0.81, 1.36)	0.91
Transient ischaemic attack			
<0.05	0.88 (0.65, 1.19)	1.01 (0.88, 1.16)	0.42
0.05−0.15	0.92 (0.78, 1.09)	0.96 (0.89, 1.03)	0.68
0.15−0.25	1 (reference)	1 (reference)	
0.25−0.35	0.86 (0.68, 1.07)	1.01 (0.92, 1.12)	0.18
≥ 0.35	0.97 (0.77, 1.22)	1.01 (0.91, 1.12)	0.79
Ischaemic stroke			
<0.05	0.78 (0.51, 1.20)	1.06 (0.91, 1.24)	0.19
0.05−0.15	1.06 (0.85, 1.32)	0.99 (0.91, 1.08)	0.61
0.15−0.25	1 (reference)	1 (reference)	
0.25−0.35	0.98 (0.72, 1.31)	1.08 (0.96, 1.20)	0.54
≥ 0.35	0.98 (0.72, 1.35)	0.99 (0.88, 1.12)	0.96
Subarachnoid haemorrhage			
<0.05	3.36 (1.44, 7.83) **	1.08 (0.72, 1.62)	0.018
0.05−0.15	1.64 (0.86, 3.11)	1.15 (0.93, 1.43)	0.31
0.15−0.25	1 (reference)	1 (reference)	
0.25−0.35	0.58 (0.19, 1.76)	1.09 (0.81, 1.45)	0.29
≥ 0.35	1.56 (0.67, 3.61)	1.05 (0.77, 1.44)	0.39
Intracerebral haemorrhage			
<0.05	1.86 (0.94, 3.66)	1.27 (0.98, 1.64)	0.3
0.05−0.15	1.27 (0.77, 2.07)	1.02 (0.87, 1.20)	0.42
0.15−0.25	1 (reference)	1 (reference)	
0.25−0.35	1.19 (0.62, 2.29)	0.78 (0.62, 0.98) *	0.23
≥ 0.35	1.30 (0.67, 2.55)	1.00 (0.80, 1.25)	0.46
Peripheral arterial disease			
<0.05	0.63 (0.44, 0.89) **	0.92 (0.79, 1.07)	0.047
0.05−0.15	0.69 (0.59, 0.82) ***	0.97 (0.89, 1.04)	0.0005
0.15−0.25	1 (reference)	1 (reference)	
0.25−0.35	0.83 (0.68, 1.03)	1.07 (0.97, 1.18)	0.031
≥ 0.35	0.93 (0.75, 1.14)	1.14 (1.03, 1.25) **	0.085
Abdominal aortic aneurysm			
<0.05	1.10 (0.62, 1.94)	0.72 (0.52, 0.99) *	0.2
0.05−0.15	1.10 (0.79, 1.53)	0.91 (0.79, 1.06)	0.3
0.15−0.25	1 (reference)	1 (reference)	
0.25−0.35	1.11 (0.75, 1.66)	1.05 (0.88, 1.25)	0.8
≥ 0.35	1.18 (0.79, 1.76)	1.02 (0.85, 1.24)	0.54

Supplementary Figure 9. Multivariable adjusted association of lymphocyte quintiles with different initial presentations of cardiovascular disease, by time after measurement

Hazard ratios are adjusted for age, sex, deprivation, ethnicity, smoking, diabetes, systolic blood pressure, body mass index, total cholesterol, HDL cholesterol, eGFR, atrial fibrillation, autoimmune conditions, inflammatory bowel disease, COPD, cancer, statin use, blood pressure medication and acute conditions at the time of blood testing. P values * < 0.05, ** < 0.01, *** < 0.001

Initial presentation	*First 6 months* Hazard ratio (95% CI)	*6 months onwards* Hazard ratio (95% CI)	*P value for interaction*
Stable angina			
<1.45	0.93 (0.80, 1.08)	1.04 (0.96, 1.12)	0.21
1.45−1.85	1.12 (0.98, 1.28)	1.00 (0.93, 1.07)	0.14
1.85−2.15	1 (reference)	1 (reference)	
2.15−2.55	1.10 (0.96, 1.26)	1.03 (0.95, 1.11)	0.41
≥ 2.55	1.20 (1.05, 1.37) **	1.05 (0.98, 1.13)	0.089
Unstable angina			
<1.45	1.05 (0.74, 1.47)	0.98 (0.85, 1.13)	0.74
1.45−1.85	1.02 (0.74, 1.40)	1.03 (0.91, 1.17)	0.96
1.85−2.15	1 (reference)	1 (reference)	
2.15−2.55	0.98 (0.70, 1.37)	1.18 (1.04, 1.35) *	0.3
≥ 2.55	1.11 (0.81, 1.52)	1.16 (1.02, 1.31) *	0.8
Non−fatal myocardial infarction			
<1.45	1.11 (0.88, 1.39)	1.00 (0.92, 1.10)	0.44
1.45−1.85	1.06 (0.85, 1.31)	1.01 (0.93, 1.10)	0.71
1.85−2.15	1 (reference)	1 (reference)	
2.15−2.55	1.04 (0.82, 1.31)	1.01 (0.93, 1.11)	0.86
≥ 2.55	1.03 (0.83, 1.29)	1.13 (1.04, 1.23) **	0.45
Unheralded coronary death			
<1.45	1.65 (1.22, 2.24) **	1.20 (1.06, 1.36) **	0.057
1.45−1.85	1.16 (0.84, 1.60)	1.01 (0.89, 1.14)	0.42
1.85−2.15	1 (reference)	1 (reference)	
2.15−2.55	1.05 (0.73, 1.50)	0.93 (0.81, 1.07)	0.56
≥ 2.55	1.03 (0.73, 1.46)	0.90 (0.79, 1.03)	0.47
Heart failure			
<1.45	2.25 (1.90, 2.67) ***	1.42 (1.30, 1.56) ***	<0.0001
1.45−1.85	1.39 (1.16, 1.67) ***	1.14 (1.04, 1.25) **	0.061
1.85−2.15	1 (reference)	1 (reference)	
2.15−2.55	1.02 (0.82, 1.26)	1.01 (0.91, 1.13)	0.96
≥ 2.55	0.94 (0.76, 1.16)	0.97 (0.88, 1.08)	0.77
Ventricular arrhythmia or sudden cardiac death			
<1.45	1.83 (1.02, 3.28) *	1.12 (0.88, 1.43)	0.12
1.45−1.85	0.96 (0.51, 1.81)	0.84 (0.66, 1.07)	0.69
1.85−2.15	1 (reference)	1 (reference)	
2.15−2.55	0.67 (0.31, 1.42)	0.76 (0.58, 0.99) *	0.75
≥ 2.55	0.82 (0.42, 1.63)	1.04 (0.81, 1.33)	0.53
Transient ischaemic attack			
<1.45	1.09 (0.88, 1.36)	1.11 (1.01, 1.23) *	0.89
1.45−1.85	1.01 (0.81, 1.25)	1.01 (0.92, 1.11)	0.98
1.85−2.15	1 (reference)	1 (reference)	
2.15−2.55	1.08 (0.85, 1.37)	1.02 (0.92, 1.13)	0.67
≥ 2.55	1.18 (0.94, 1.48)	1.02 (0.93, 1.13)	0.27
Ischaemic stroke			
<1.45	1.17 (0.87, 1.56)	1.07 (0.95, 1.19)	0.58
1.45−1.85	1.10 (0.82, 1.48)	0.99 (0.89, 1.11)	0.51
1.85−2.15	1 (reference)	1 (reference)	
2.15−2.55	1.06 (0.76, 1.47)	0.93 (0.82, 1.05)	0.46
≥ 2.55	1.06 (0.78, 1.46)	0.99 (0.88, 1.11)	0.68
Subarachnoid haemorrhage			
<1.45	1.33 (0.55, 3.21)	1.30 (0.98, 1.72)	0.96
1.45−1.85	1.84 (0.84, 4.03)	0.88 (0.67, 1.16)	0.081
1.85−2.15	1 (reference)	1 (reference)	
2.15−2.55	0.67 (0.24, 1.87)	0.84 (0.62, 1.12)	0.68
≥ 2.55	1.63 (0.73, 3.64)	1.04 (0.79, 1.37)	0.3
Intracerebral haemorrhage			
<1.45	0.78 (0.45, 1.34)	1.58 (1.28, 1.94) ***	0.017
1.45−1.85	0.67 (0.38, 1.19)	1.19 (0.96, 1.47)	0.065
1.85−2.15	1 (reference)	1 (reference)	
2.15−2.55	0.45 (0.21, 0.93) *	1.14 (0.91, 1.44)	0.017
≥ 2.55	0.88 (0.49, 1.58)	1.04 (0.82, 1.32)	0.6
Peripheral arterial disease			
<1.45	0.91 (0.72, 1.16)	1.14 (1.03, 1.27) *	0.094
1.45−1.85	0.92 (0.73, 1.15)	1.01 (0.91, 1.11)	0.48
1.85−2.15	1 (reference)	1 (reference)	
2.15−2.55	0.96 (0.76, 1.22)	1.08 (0.98, 1.20)	0.37
≥ 2.55	1.33 (1.08, 1.64) **	1.25 (1.13, 1.37) ***	0.58
Abdominal aortic aneurysm			
<1.45	1.16 (0.77, 1.77)	0.76 (0.63, 0.92) **	0.067
1.45−1.85	1.08 (0.71, 1.65)	0.79 (0.66, 0.95) *	0.18
1.85−2.15	1 (reference)	1 (reference)	
2.15−2.55	1.19 (0.76, 1.87)	0.83 (0.69, 1.01)	0.16
≥ 2.55	1.20 (0.78, 1.85)	0.84 (0.70, 1.01)	0.14

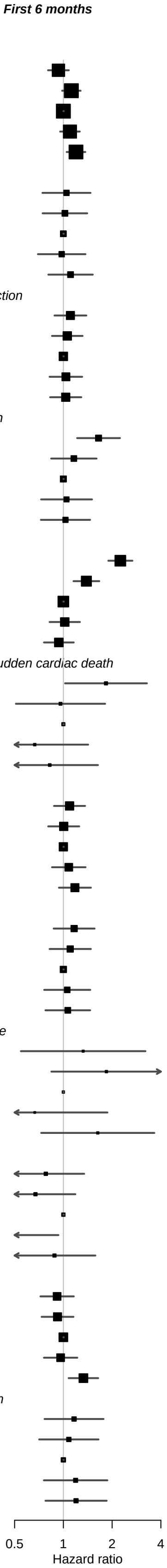

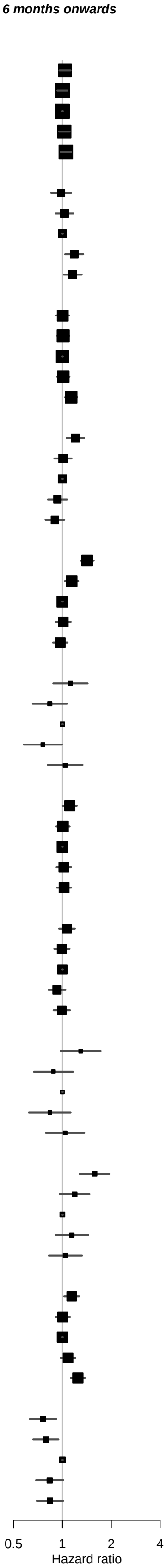

Supplementary Figure 10. Association of eosinophil categories with different initial presentations of cardiovascular disease, by patient state at the time of blood sampling

Hazard ratios are adjusted for age, sex, deprivation, ethnicity, smoking, diabetes, systolic blood pressure, body mass index, total cholesterol, HDL cholesterol, eGFR, atrial fibrillation, autoimmune conditions, inflammatory bowel disease, COPD, cancer, statin use, blood pressure medication and acute conditions at the time of blood testing.

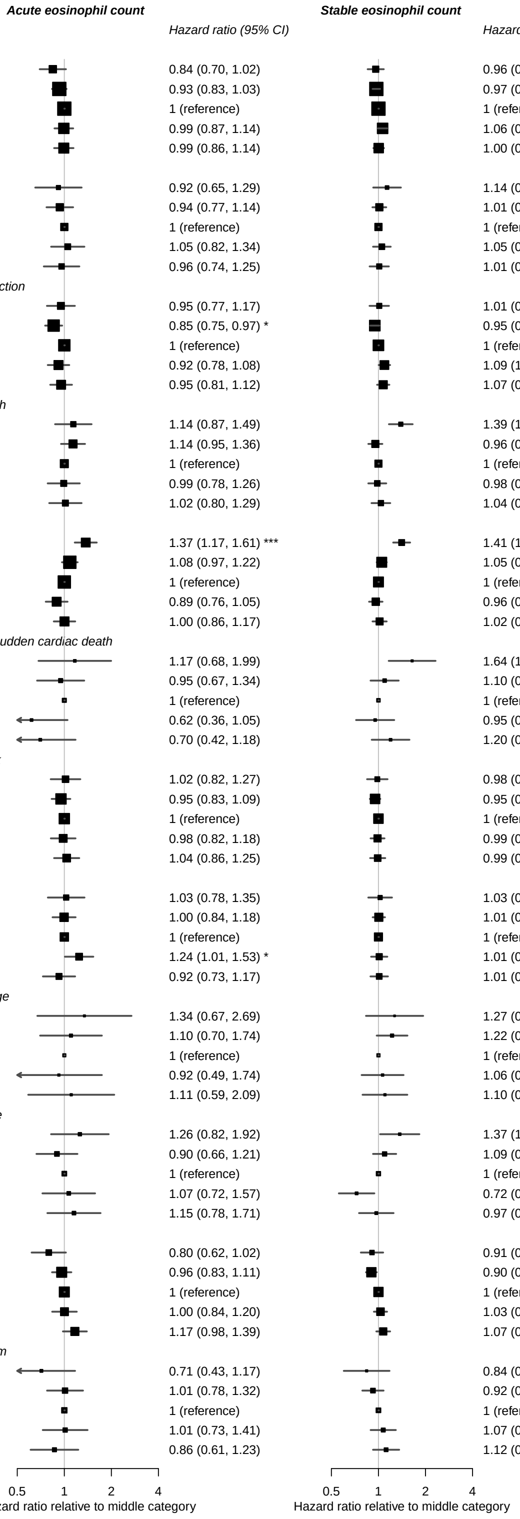

Initial presentation	*Acute eosinophil count* Hazard ratio (95% CI)	*Stable eosinophil count* Hazard ratio (95% CI)	*P value for interaction*
Stable angina			
<0.05	0.84 (0.70, 1.02)	0.96 (0.85, 1.08)	0.26
0.05−0.15	0.93 (0.83, 1.03)	0.97 (0.92, 1.02)	0.48
0.15−0.25	1 (reference)	1 (reference)	
0.25−0.35	0.99 (0.87, 1.14)	1.06 (0.99, 1.14)	0.39
≥ 0.35	0.99 (0.86, 1.14)	1.00 (0.93, 1.08)	0.89
Unstable angina			
<0.05	0.92 (0.65, 1.29)	1.14 (0.93, 1.39)	0.29
0.05−0.15	0.94 (0.77, 1.14)	1.01 (0.92, 1.12)	0.48
0.15−0.25	1 (reference)	1 (reference)	
0.25−0.35	1.05 (0.82, 1.34)	1.05 (0.92, 1.20)	1
≥ 0.35	0.96 (0.74, 1.25)	1.01 (0.88, 1.17)	0.73
Non−fatal myocardial infarction			
<0.05	0.95 (0.77, 1.17)	1.01 (0.88, 1.17)	0.63
0.05−0.15	0.85 (0.75, 0.97) *	0.95 (0.88, 1.02)	0.16
0.15−0.25	1 (reference)	1 (reference)	
0.25−0.35	0.92 (0.78, 1.08)	1.09 (1.00, 1.19) *	0.058
≥ 0.35	0.95 (0.81, 1.12)	1.07 (0.98, 1.17)	0.21
Unheralded coronary death			
<0.05	1.14 (0.87, 1.49)	1.39 (1.17, 1.65) ***	0.23
0.05−0.15	1.14 (0.95, 1.36)	0.96 (0.86, 1.06)	0.095
0.15−0.25	1 (reference)	1 (reference)	
0.25−0.35	0.99 (0.78, 1.26)	0.98 (0.86, 1.12)	0.95
≥ 0.35	1.02 (0.80, 1.29)	1.04 (0.90, 1.19)	0.89
Heart failure			
<0.05	1.37 (1.17, 1.61) ***	1.41 (1.24, 1.60) ***	0.8
0.05−0.15	1.08 (0.97, 1.22)	1.05 (0.97, 1.13)	0.64
0.15−0.25	1 (reference)	1 (reference)	
0.25−0.35	0.89 (0.76, 1.05)	0.96 (0.87, 1.06)	0.44
≥ 0.35	1.00 (0.86, 1.17)	1.02 (0.92, 1.13)	0.88
Ventricular arrhythmia or sudden cardiac death			
<0.05	1.17 (0.68, 1.99)	1.64 (1.16, 2.32) **	0.29
0.05−0.15	0.95 (0.67, 1.34)	1.10 (0.89, 1.35)	0.48
0.15−0.25	1 (reference)	1 (reference)	
0.25−0.35	0.62 (0.36, 1.05)	0.95 (0.72, 1.26)	0.16
≥ 0.35	0.70 (0.42, 1.18)	1.20 (0.91, 1.58)	0.076
Transient ischaemic attack			
<0.05	1.02 (0.82, 1.27)	0.98 (0.85, 1.14)	0.8
0.05−0.15	0.95 (0.83, 1.09)	0.95 (0.88, 1.03)	1
0.15−0.25	1 (reference)	1 (reference)	
0.25−0.35	0.98 (0.82, 1.18)	0.99 (0.89, 1.09)	0.98
≥ 0.35	1.04 (0.86, 1.25)	0.99 (0.88, 1.11)	0.68
Ischaemic stroke			
<0.05	1.03 (0.78, 1.35)	1.03 (0.86, 1.22)	0.99
0.05−0.15	1.00 (0.84, 1.18)	1.01 (0.92, 1.10)	0.93
0.15−0.25	1 (reference)	1 (reference)	
0.25−0.35	1.24 (1.01, 1.53) *	1.01 (0.90, 1.14)	0.093
≥ 0.35	0.92 (0.73, 1.17)	1.01 (0.89, 1.15)	0.52
Subarachnoid haemorrhage			
<0.05	1.34 (0.67, 2.69)	1.27 (0.83, 1.93)	0.89
0.05−0.15	1.10 (0.70, 1.74)	1.22 (0.97, 1.53)	0.69
0.15−0.25	1 (reference)	1 (reference)	
0.25−0.35	0.92 (0.49, 1.74)	1.06 (0.78, 1.45)	0.69
≥ 0.35	1.11 (0.59, 2.09)	1.10 (0.79, 1.53)	0.99
Intracerebral haemorrhage			
<0.05	1.26 (0.82, 1.92)	1.37 (1.02, 1.83) *	0.75
0.05−0.15	0.90 (0.66, 1.21)	1.09 (0.92, 1.30)	0.26
0.15−0.25	1 (reference)	1 (reference)	
0.25−0.35	1.07 (0.72, 1.57)	0.72 (0.56, 0.94) *	0.1
≥ 0.35	1.15 (0.78, 1.71)	0.97 (0.75, 1.25)	0.46
Peripheral arterial disease			
<0.05	0.80 (0.62, 1.02)	0.91 (0.77, 1.07)	0.38
0.05−0.15	0.96 (0.83, 1.11)	0.90 (0.83, 0.98) **	0.43
0.15−0.25	1 (reference)	1 (reference)	
0.25−0.35	1.00 (0.84, 1.20)	1.03 (0.94, 1.14)	0.79
≥ 0.35	1.17 (0.98, 1.39)	1.07 (0.97, 1.19)	0.41
Abdominal aortic aneurysm			
<0.05	0.71 (0.43, 1.17)	0.84 (0.60, 1.18)	0.59
0.05−0.15	1.01 (0.78, 1.32)	0.92 (0.79, 1.08)	0.56
0.15−0.25	1 (reference)	1 (reference)	
0.25−0.35	1.01 (0.73, 1.41)	1.07 (0.89, 1.29)	0.76
≥ 0.35	0.86 (0.61, 1.23)	1.12 (0.92, 1.36)	0.2

Supplementary Figure 11. Association of lymphocyte quintiles with different initial presentations of cardiovascular disease, by patient state at the time of blood sampling

Hazard ratios are adjusted for age, sex, deprivation, ethnicity, smoking, diabetes, systolic blood pressure, body mass index, total cholesterol, HDL cholesterol, eGFR, atrial fibrillation, autoimmune conditions, inflammatory bowel disease, COPD, cancer, statin use, blood pressure medication and acute conditions at the time of blood testing.

Initial presentation	Acute lymphocyte count: Hazard ratio (95% CI)	Stable lymphocyte count: Hazard ratio (95% CI)	P value for interaction
Stable angina			
<1.45	0.97 (0.84, 1.12)	1.03 (0.95, 1.11)	0.49
1.45−1.85	0.99 (0.87, 1.14)	1.04 (0.97, 1.11)	0.61
1.85−2.15	1 (reference)	1 (reference)	
2.15−2.55	0.98 (0.84, 1.14)	1.06 (0.98, 1.14)	0.36
≥ 2.55	1.07 (0.93, 1.23)	1.08 (1.01, 1.17) *	0.87
Unstable angina			
<1.45	0.99 (0.76, 1.30)	1.00 (0.86, 1.16)	0.96
1.45−1.85	1.21 (0.94, 1.56)	0.98 (0.86, 1.12)	0.15
1.85−2.15	1 (reference)	1 (reference)	
2.15−2.55	1.05 (0.80, 1.39)	1.18 (1.03, 1.35) *	0.46
≥ 2.55	1.14 (0.88, 1.48)	1.15 (1.01, 1.32) *	0.94
Non−fatal myocardial infarction			
<1.45	0.95 (0.80, 1.12)	1.05 (0.95, 1.15)	0.32
1.45−1.85	1.02 (0.87, 1.20)	1.01 (0.93, 1.11)	0.93
1.85−2.15	1 (reference)	1 (reference)	
2.15−2.55	0.96 (0.81, 1.15)	1.03 (0.94, 1.13)	0.48
≥ 2.55	1.04 (0.88, 1.23)	1.14 (1.05, 1.25) **	0.3
Unheralded coronary death			
<1.45	1.34 (1.07, 1.67) *	1.24 (1.09, 1.42) **	0.58
1.45−1.85	1.09 (0.86, 1.37)	1.01 (0.89, 1.15)	0.59
1.85−2.15	1 (reference)	1 (reference)	
2.15−2.55	0.95 (0.73, 1.25)	0.94 (0.82, 1.09)	0.95
≥ 2.55	0.97 (0.75, 1.25)	0.90 (0.78, 1.04)	0.61
Heart failure			
<1.45	1.64 (1.41, 1.91) ***	1.60 (1.45, 1.76) ***	0.77
1.45−1.85	1.24 (1.06, 1.46) **	1.17 (1.06, 1.29) **	0.51
1.85−2.15	1 (reference)	1 (reference)	
2.15−2.55	1.18 (0.99, 1.41)	0.96 (0.86, 1.07)	0.057
≥ 2.55	0.97 (0.81, 1.16)	0.96 (0.87, 1.08)	0.96
Ventricular arrhythmia or sudden cardiac death			
<1.45	1.00 (0.64, 1.55)	1.30 (1.00, 1.68) *	0.31
1.45−1.85	0.94 (0.61, 1.46)	0.81 (0.62, 1.06)	0.58
1.85−2.15	1 (reference)	1 (reference)	
2.15−2.55	0.49 (0.27, 0.88) *	0.83 (0.62, 1.10)	0.11
≥ 2.55	0.89 (0.56, 1.43)	1.05 (0.81, 1.37)	0.54
Transient ischaemic attack			
<1.45	1.21 (1.01, 1.45) *	1.08 (0.98, 1.20)	0.31
1.45−1.85	1.12 (0.94, 1.35)	0.98 (0.89, 1.08)	0.19
1.85−2.15	1 (reference)	1 (reference)	
2.15−2.55	1.12 (0.91, 1.37)	1.01 (0.90, 1.12)	0.37
≥ 2.55	1.14 (0.93, 1.38)	1.03 (0.92, 1.14)	0.36
Ischaemic stroke			
<1.45	1.07 (0.87, 1.33)	1.08 (0.96, 1.22)	0.95
1.45−1.85	0.99 (0.79, 1.23)	1.01 (0.90, 1.13)	0.87
1.85−2.15	1 (reference)	1 (reference)	
2.15−2.55	0.90 (0.70, 1.15)	0.95 (0.84, 1.08)	0.68
≥ 2.55	1.04 (0.83, 1.31)	0.98 (0.87, 1.11)	0.66
Subarachnoid haemorrhage			
<1.45	1.17 (0.66, 2.05)	1.33 (0.98, 1.81)	0.68
1.45−1.85	0.68 (0.37, 1.26)	1.04 (0.78, 1.39)	0.22
1.85−2.15	1 (reference)	1 (reference)	
2.15−2.55	0.75 (0.39, 1.44)	0.84 (0.61, 1.15)	0.75
≥ 2.55	1.10 (0.63, 1.93)	1.09 (0.81, 1.45)	0.97
Intracerebral haemorrhage			
<1.45	1.22 (0.84, 1.76)	1.56 (1.24, 1.95) ***	0.26
1.45−1.85	1.05 (0.72, 1.55)	1.13 (0.90, 1.42)	0.75
1.85−2.15	1 (reference)	1 (reference)	
2.15−2.55	0.98 (0.63, 1.52)	1.06 (0.82, 1.37)	0.76
≥ 2.55	0.97 (0.63, 1.48)	1.04 (0.81, 1.34)	0.78
Peripheral arterial disease			
<1.45	1.12 (0.93, 1.35)	1.09 (0.97, 1.22)	0.78
1.45−1.85	0.96 (0.79, 1.16)	1.00 (0.90, 1.11)	0.72
1.85−2.15	1 (reference)	1 (reference)	
2.15−2.55	1.10 (0.90, 1.34)	1.05 (0.94, 1.17)	0.7
≥ 2.55	1.11 (0.92, 1.34)	1.30 (1.18, 1.44) ***	0.14
Abdominal aortic aneurysm			
<1.45	0.83 (0.59, 1.17)	0.82 (0.67, 1.00)	0.94
1.45−1.85	0.92 (0.66, 1.30)	0.80 (0.66, 0.97) *	0.47
1.85−2.15	1 (reference)	1 (reference)	
2.15−2.55	1.07 (0.74, 1.53)	0.83 (0.67, 1.01)	0.23
≥ 2.55	0.92 (0.64, 1.31)	0.88 (0.72, 1.06)	0.82

Hazard ratio relative to middle category (axis: 0.5, 1, 2, 4) — Acute lymphocyte count; Stable lymphocyte count

Supplementary Figure 12. Linear association of eosinophil counts with different initial presentations of cardiovascular disease, by patient state at the time of blood sampling

Hazard ratios are adjusted for age, sex, deprivation, ethnicity, smoking, diabetes, systolic blood pressure, body mass index, total cholesterol, HDL cholesterol, eGFR, atrial fibrillation, autoimmune conditions, inflammatory bowel disease, COPD, cancer, statin use, blood pressure medication and acute conditions at the time of blood testing.

Initial presentation	*Hazard ratio (95% CI)*	*P value for interaction*
Stable angina		
Acute	1.00 (0.98, 1.02)	0.7
Stable	1.01 (0.99, 1.02)	
Unstable angina		
Acute	0.99 (0.95, 1.03)	0.91
Stable	0.99 (0.97, 1.02)	
Coronary disease not further specified		
Acute	0.99 (0.96, 1.03)	0.27
Stable	1.01 (0.99, 1.03)	
Non−fatal myocardial infarction		
Acute	1.00 (0.98, 1.03)	0.19
Stable	1.02 (1.01, 1.04) **	
Unheralded coronary death		
Acute	0.98 (0.94, 1.02)	0.4
Stable	1.00 (0.97, 1.02)	
Heart failure		
Acute	0.96 (0.94, 0.99) *	0.35
Stable	0.98 (0.96, 1.00)	
Ventricular arrhythmia or sudden cardiac death		
Acute	0.95 (0.86, 1.04)	0.5
Stable	0.99 (0.93, 1.04)	
Transient ischaemic attack		
Acute	1.00 (0.97, 1.03)	0.8
Stable	1.00 (0.98, 1.02)	
Ischaemic stroke		
Acute	0.99 (0.96, 1.03)	0.91
Stable	1.00 (0.97, 1.02)	
Stroke not further specified		
Acute	1.01 (0.98, 1.04)	1
Stable	1.01 (0.99, 1.03)	
Subarachnoid haemorrhage		
Acute	0.96 (0.86, 1.09)	0.9
Stable	0.96 (0.89, 1.02)	
Intracerebral haemorrhage		
Acute	1.01 (0.95, 1.06)	0.41
Stable	0.97 (0.93, 1.03)	
Peripheral arterial disease		
Acute	1.02 (1.00, 1.04) *	0.84
Stable	1.03 (1.01, 1.04) **	
Abdominal aortic aneurysm		
Acute	0.99 (0.93, 1.04)	0.44
Stable	1.01 (0.98, 1.04)	

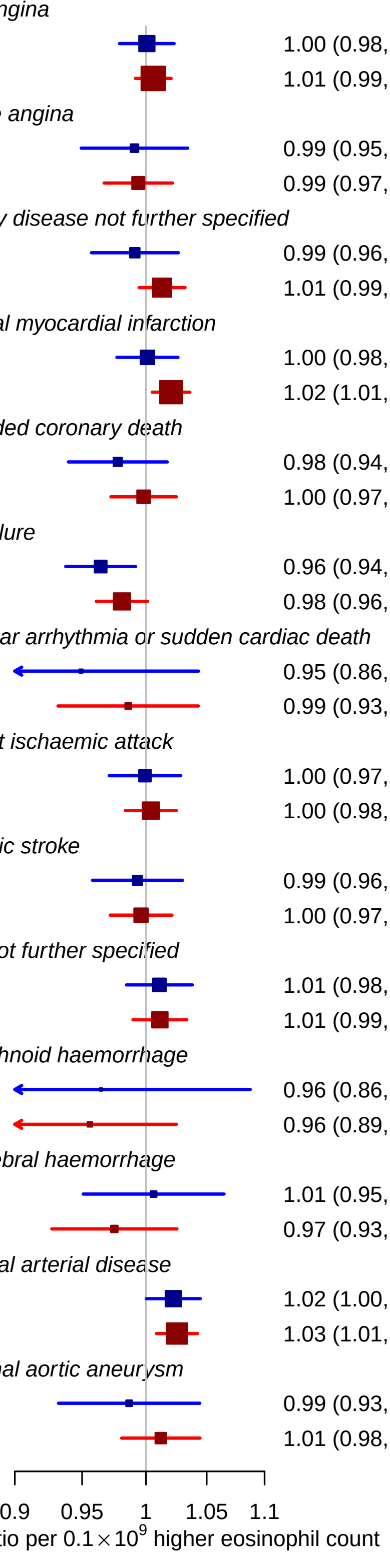

Supplementary Figure 13. Linear association of eosinophil counts with different initial presentations of cardiovascular disease, by time since blood sampling

Hazard ratios are adjusted for age, sex, deprivation, ethnicity, smoking, diabetes, systolic blood pressure, body mass index, total cholesterol, HDL cholesterol, eGFR, atrial fibrillation, autoimmune conditions, inflammatory bowel disease, COPD, cancer, statin use, blood pressure medication and acute conditions at the time of blood testing.

Initial presentation	*Hazard ratio (95% CI)*	*P value for interaction*
Stable angina		
First 6 months	1.01 (0.98, 1.03)	0.74
6 months onwards	1.00 (0.99, 1.02)	
Unstable angina		
First 6 months	0.94 (0.87, 1.01)	0.12
6 months onwards	1.00 (0.98, 1.02)	
Coronary disease not further specified		
First 6 months	0.98 (0.94, 1.02)	0.17
6 months onwards	1.01 (1.00, 1.03)	
Non−fatal myocardial infarction		
First 6 months	0.97 (0.93, 1.02)	0.057
6 months onwards	1.02 (1.01, 1.03) **	
Unheralded coronary death		
First 6 months	0.97 (0.92, 1.03)	0.5
6 months onwards	1.00 (0.97, 1.02)	
Heart failure		
First 6 months	0.89 (0.86, 0.93) ***	<0.0001
6 months onwards	1.00 (0.98, 1.02)	
Ventricular arrhythmia or sudden cardiac death		
First 6 months	0.90 (0.78, 1.04)	0.24
6 months onwards	0.99 (0.94, 1.04)	
Transient ischaemic attack		
First 6 months	1.01 (0.97, 1.05)	0.85
6 months onwards	1.00 (0.98, 1.02)	
Ischaemic stroke		
First 6 months	1.00 (0.95, 1.06)	0.79
6 months onwards	0.99 (0.97, 1.02)	
Stroke not further specified		
First 6 months	1.02 (0.99, 1.05)	0.59
6 months onwards	1.01 (0.99, 1.03)	
Subarachnoid haemorrhage		
First 6 months	0.89 (0.73, 1.08)	0.43
6 months onwards	0.97 (0.91, 1.03)	
Intracerebral haemorrhage		
First 6 months	0.97 (0.85, 1.10)	0.73
6 months onwards	0.99 (0.95, 1.03)	
Peripheral arterial disease		
First 6 months	1.03 (1.00, 1.06) *	0.58
6 months onwards	1.02 (1.01, 1.04) **	
Abdominal aortic aneurysm		
First 6 months	0.98 (0.91, 1.05)	0.41
6 months onwards	1.01 (0.98, 1.04)	

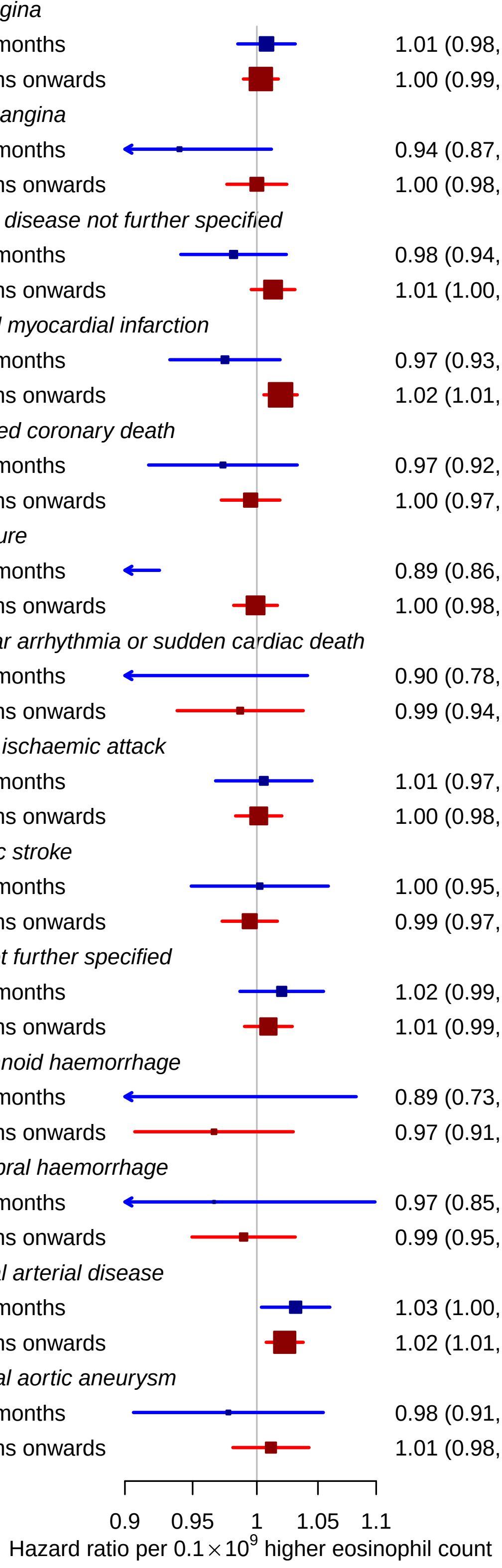

Supplementary Figure 14. Binned scatterplot showing difference between two consecutive eosinophil counts taken when a patient was clinically 'stable'

N = 395 133. Time between blood tests: median 1.37 years, interquartile range 0.57–2.74 years. Correlation coefficient = 0.597. Standard deviation of differences = 0.14×10^9/L. Mean change over time: increase of 0.0054×10^9/L (95% CI 0.0029–0.0079×10^9/L) per year. The solid line is lowess smoothed mean and the dotted lines are lowess smoothed 2.5% and 97.5% centiles.

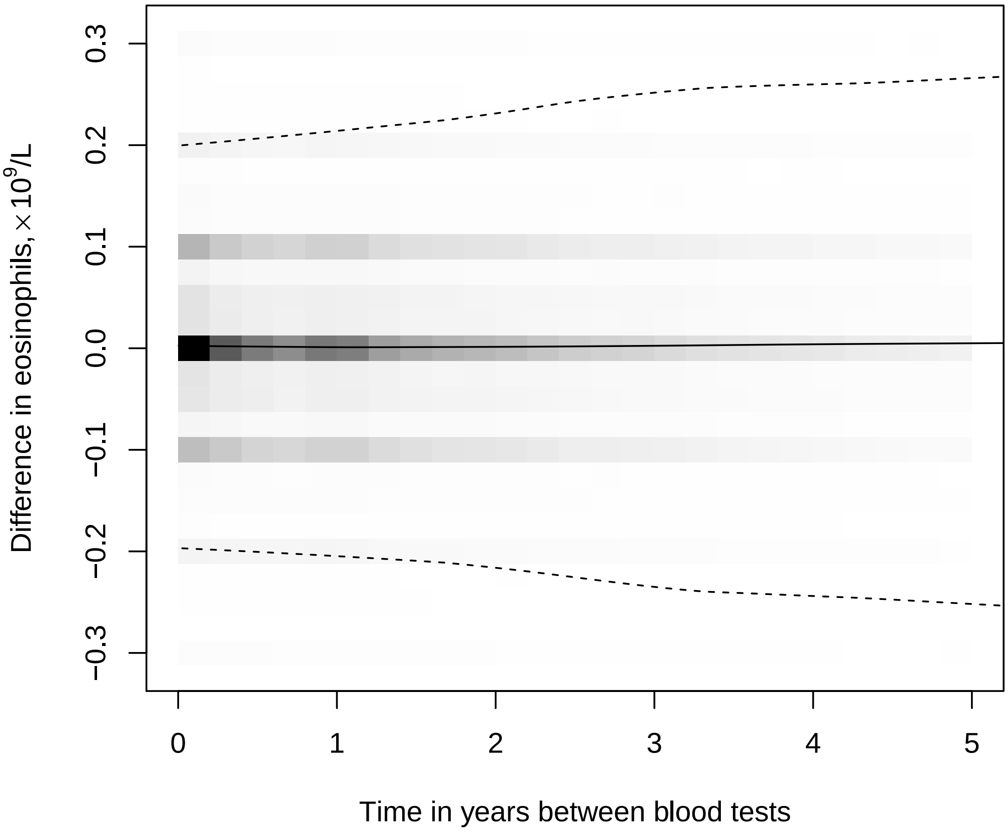

Supplementary Figure 15. Binned scatterplot showing difference between two consecutive lymphocyte counts taken when a patient was clinically 'stable'

N = 395 133. Time between blood tests: median 1.37 years, interquartile range 0.57–2.74 years. Correlation coefficient = 0.706. Standard deviation of differences = 0.59×10^9/L. Mean change over time: increase of 0.0036×10^9/L (95% CI $0.0025–0.0046\times10^9$/L) per year. The solid line is lowess smoothed mean and the dotted lines are lowess smoothed 2.5% and 97.5% centiles.

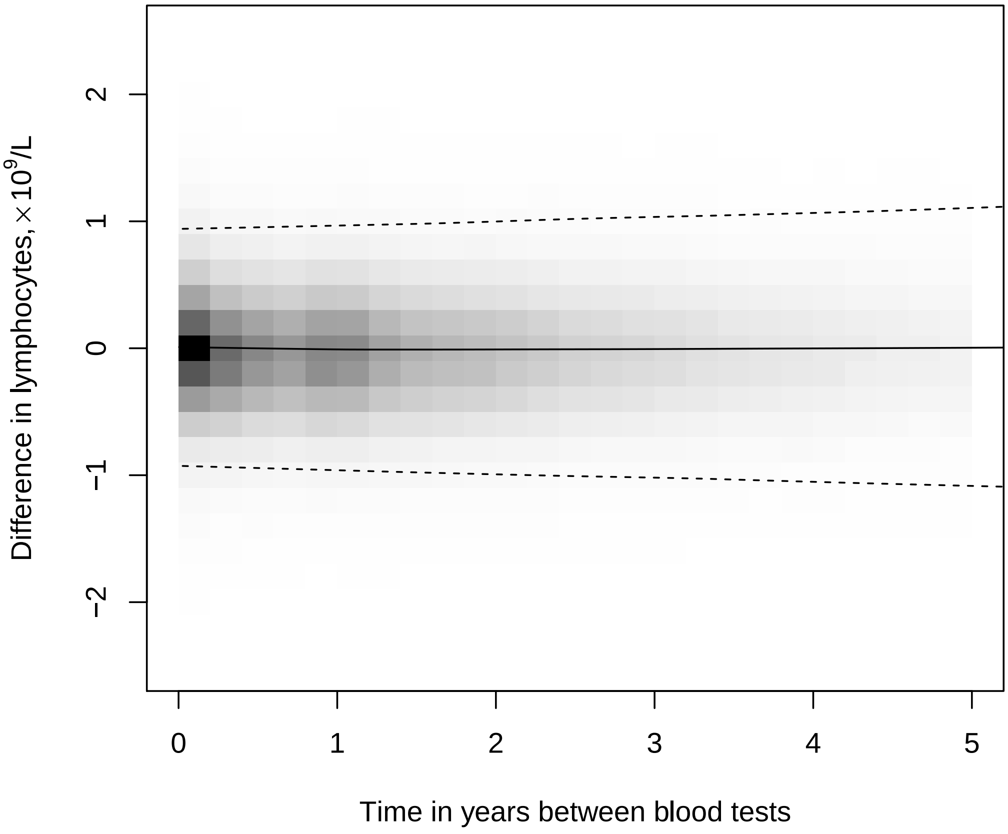

Supplementary References

1. Denaxas SC, George J, Herrett E, et al. Data Resource Profile: Cardiovascular disease research using linked bespoke studies and electronic health records (CALIBER). Int J Epidemiol. 2012 Dec;41(6):1625–1638. http://dx.doi.org/10.1093/ije/dys188.

2. Herrett E, Gallagher AM, Bhaskaran K, et al. Data Resource Profile: Clinical Practice Research Datalink (CPRD). Int J Epidemiol. 2015 Jun;44(3):827–836. http://dx.doi.org/10.1093/ije/dyv098.

3. Herrett E, Smeeth L, Walker L, et al. The Myocardial Ischaemia National Audit Project (MINAP). Heart. 2010 Aug;96(16):1264–1267. http://dx.doi.org/10.1136/hrt.2009.192328.

4. Herrett E, Shah AD, Boggon R, et al. Completeness and diagnostic validity of recording acute myocardial infarction events in primary care, hospital care, disease registry, and national mortality records: cohort study. BMJ. 2013 5;346:f2350.

5. Shah AD, Bartlett JW, Carpenter JR, et al. Comparison of Random Forest and parametric imputation models when imputing missing data using MICE: a CALIBER study. Am J Epidemiol. 2014 Mar;179(6):764–774.

6. Jarvik G, Crosslin D. eMERGE Network Supplemental Genotyping Project – Phenotype Description & Specifications for White Blood Cell Count. eMERGE Network Supplemental Genotyping Project; 2012. https://www.phekb.org/phenotype/19.

7. Gottesman O, Kuivaniemi H, Tromp G, et al. The Electronic Medical Records and Genomics (eMERGE) Network: past, present, and future. Genet Med. 2013 Oct;15(10):761–771. http://dx.doi.org/10.1038/gim.2013.72.

8. Rubin DB. Multiple imputation after 18+ years. J Am Stat Assoc. 1996 Jun;91:473–489.

9. Prentice RL, Szatrowski TP, Fujikura T, et al. Leukocyte counts and coronary heart disease in a Japanese cohort. Am J Epidemiol. 1982;116(3):496–509. http://aje.oxfordjournals.org/cgi/content/abstract/116/3/4.

10. Sweetnam PM, Thomas HF, Yarnell JW, et al. Total and differential leukocyte counts as predictors of ischemic heart disease: the Caerphilly and Speedwell studies. Am J Epidemiol. 1997;145(5):416–21.

11. Olivares R, Ducimetière P, Claude JR. Monocyte Count: A Risk Factor for Coronary Heart Disease? Am J Epidemiol. 1993;137(1):49–53.

12. Adamsson Eryd S, Smith JG, Melander O, et al. Incidence of coronary events and case fatality rate in relation to blood lymphocyte and neutrophil counts. Arterioscler Thromb Vasc Biol. 2012;32(2):533–9. http://atvb.ahajournals.org/content/32/2/533.long.

13. Bekwelem W, Lutsey PL, Loehr LR, et al. White Blood Cell Count, C-Reactive Protein, and Incident Heart Failure in the Atherosclerosis Risk in Communities (ARIC) Study. Ann Epidemiol. 2011;21(10):739–748.

14. Zia E, Melander O, Bjorkbacka H, et al. Total and differential leucocyte counts in relation to incidence of stroke subtypes and mortality: A prospective cohort study. J Intern Med. 2012;272(3):298–304.

15. Pfister R, Sharp SJ, Luben R, et al. Differential white blood cell count and incident heart failure in men and women in the EPIC-Norfolk study. Eur Heart J. 2012;33(4):523–30.

16. Gillum RF, Mussolino ME, Madans JH. Counts of neutrophils, lymphocytes, and monocytes, cause-specific mortality and coronary heart disease: The NHANES-I epidemiologic follow-up study. Ann Epidemiol. 2005;15(4):266–271.

17. Wheeler JG, Mussolino ME, Gillum RF, et al. Associations between differential leucocyte count and incident coronary heart disease: 1764 incident cases from seven prospective studies of 30,374 individuals. Eur Heart J. 2004 Aug;25(15):1287–1292. http://dx.doi.org/10.1016/j.ehj.2004.05.002.

18. Prentice RL, Szatrowski TP, Kato H, et al. Leukocyte counts and cerebrovascular disease. J Chronic Dis. 1982;35(9):703–714.

19. Karino S, Willcox BJ, Fong K, et al. Total and differential white blood cell counts predict eight-year incident coronary heart disease in elderly Japanese-American men: the Honolulu Heart Program. Atherosclerosis. 2015 Feb;238(2):153–158. http://dx.doi.org/10.1016/j.atherosclerosis.2014.12.003.

20. Shah N, Parikh V, Patel N, et al. Neutrophil lymphocyte ratio significantly improves the Framingham risk score in prediction of coronary heart disease mortality: Insights from the National Health and Nutrition Examination Survey-III. Int J Cardiol. 2014;171(3):390–397.
